# FormulationBCS: A Machine Learning Platform Based on Diverse Molecular Representations for Biopharmaceutical Classification System (BCS) Class Prediction

**DOI:** 10.1021/acs.molpharmaceut.4c00946

**Published:** 2024-12-08

**Authors:** Zheng Wu, Nannan Wang, Zhuyifan Ye, Huanle Xu, Ging Chan, Defang Ouyang

**Affiliations:** a Institute of Chinese Medical Sciences (ICMS), State Key Laboratory of Quality Research in Chinese Medicine, University of Macau, Macau 999078, China; b Faculty of Applied Sciences, Macao Polytechnic University, Macau 999078, China; c Faculty of Science and Technology, University of Macau, Macau 999078, China; d Department of Public Health and Medicinal Administration, Faculty of Health Sciences (FHS), University of Macau, Macau 999078, China

**Keywords:** BCS prediction, machine learning, artificial intelligence platform, preformulation, solubility, permeability

## Abstract

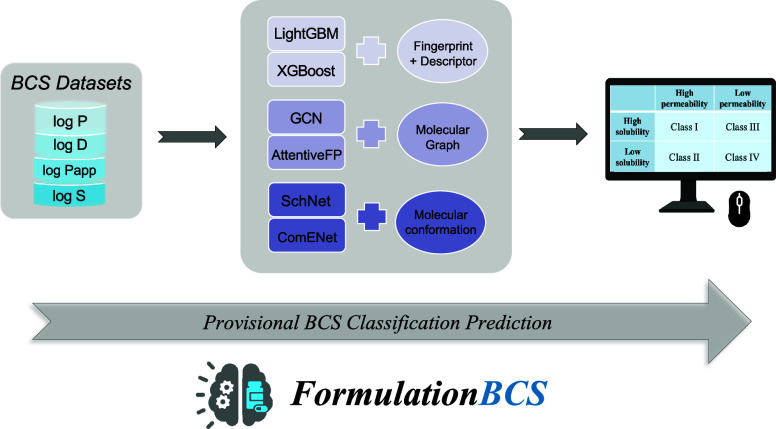

The Biopharmaceutics Classification System (BCS) has facilitated biowaivers and played a significant role in enhancing drug regulation and development efficiency. However, the productivity of measuring the key discriminative properties of BCS, solubility and permeability, still requires improvement, limiting high-throughput applications of BCS, which is essential for evaluating drug candidate developability and guiding formulation decisions in the early stages of drug development. In recent years, advancements in machine learning (ML) and molecular characterization have revealed the potential of quantitative structure–performance relationships (QSPR) for rapid and accurate *in silico* BCS classification. The present study aims to develop a web platform for high-throughput BCS classification based on high-performance ML models. Initially, four data sets of BCS-related molecular properties: log *S*, log *P*, log *D*, and log *P*_app_ were curated. Subsequently, 6 ML algorithms or deep learning frameworks were employed to construct models, with diverse molecular representations ranging from one-dimensional molecular fingerprints, descriptors, and molecular graphs to three-dimensional molecular spatial coordinates. By comparing different combinations of molecular representations and learning algorithms, LightGBM exhibited excellent performance in solubility prediction, with an *R*^2^ of 0.84; AttentiveFP outperformed others in permeability prediction, with *R*^2^ values of 0.96 and 0.76 for log *P* and log *D*, respectively; and XGBoost was the most accurate for log *P*_app_ prediction, with an *R*^2^ of 0.71. When externally validated on a marketed drug BCS category data set, the best-performing models achieved classification accuracies of over 77 and 73% for solubility and permeability, respectively. Finally, the well-trained models were embedded into the first ML-based BCS class prediction web platform (x f), enabling pharmaceutical scientists to quickly determine the BCS category of candidate drugs, which will aid in the high-throughput BCS assessment for candidate drugs during the preformulation stage, thereby promoting reduced risk and enhanced efficiency in drug development and regulation.

## 1 Introduction

**Figure 1:**
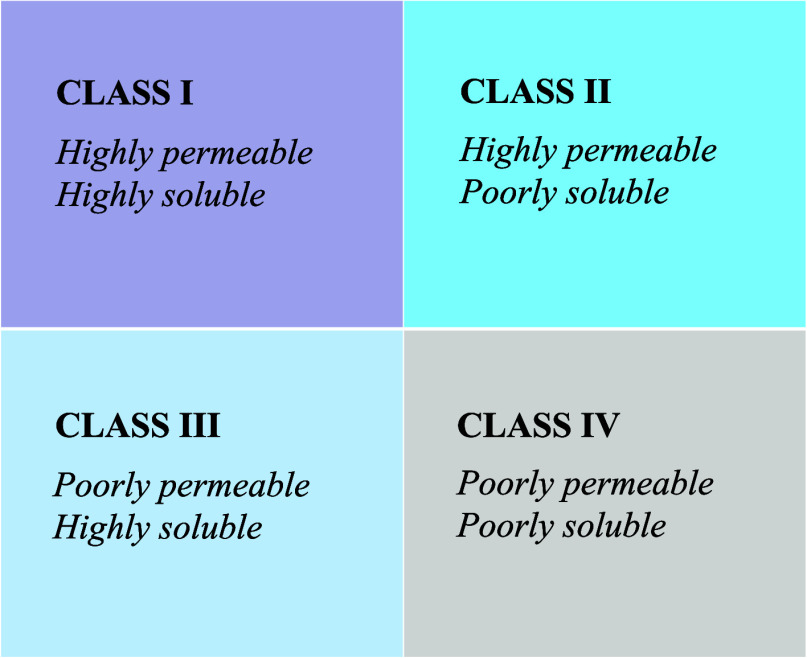
Biopharmaceutical classification system.

The Biopharmaceutics Classification System (BCS), proposed by Amidon et al.^1^ in 1995, was designed for oral immediate-release (IR) solid drugs to decrease regulatory constraints and streamline the drug development and approval processes. The BCS classifies small molecule drugs into four classes (Figure 1) based on solubility and intestinal permeability, the key attributes influencing oral drug absorption.^1^ Over two decades of exploration have underscored the profound influence of the BCS on the regulation and development of IR oral solid drug products. In particular, the BCS framework has paved the way for minimizing the necessity of clinical bioequivalence (BE) studies in humans. The validity and broad applicability of the BCS in the context of biowaivers have been corroborated through extensive research and practical applications.^2,3^ Therefrom, major drug regulatory agencies like the European Medicines Agency (EMA) and the U.S. Food and Drug Administration (FDA) have embraced this scientific theory, adopting it as a guideline for bioequivalence waivers.^4^ This has expedited the market entry of generic drugs, improving cost-efficiency. Such advancements are particularly crucial for medications treating diseases with significant societal impacts, such as oncology drug products, where traditional BE tests pose challenges.^5^

Despite BCS’s success in drug development and regulation, several challenges limit its application. One major issue is the complex and costly methods for determining solubility and permeability, which impede rapid high-throughput screening and the early stage developability assessment of drug candidates. Generally, the BCS defines high and low solubilities based on aqueous solubility over a pH range of 1.2–6.8 at 37 °C and the highest dosage strength; however, synthesizing enough compounds for solubility measurement can be expensive and inefficient, especially for large-scale screening or computer-aided drug design (CADD). Compared with solubility, permeability estimation is even more complicated. Although various quantitative measures of permeability exist, their accuracy remain uncertain.^6^ Absolute bioavailability or mass balance studies, along with other human pharmacokinetic studies, are considered the preferred methods for assessing drug permeability.^7^ Human effective permeability (*P*_eff_) across the jejunum membrane is also regarded as one of the most reliable methods.^8^ However, these approaches come with high execution costs, ethical concerns, and low throughput and are unsuitable for routine use in drug development. Alternative methods, such as *in vitro* permeability (log *P*_app_) in Caco-2 cell cultures and lipophilicity-based approaches (log *P* and log *D*), are also commonly used but face challenges relating to measurement accuracy and their ability to reflect true permeability. Consequently, even after 30 years, the number of drugs with well-defined BCS classifications remains limited,^9^ hindering the BCS from fully realizing its potential in advancing drug development.

Machine learning (ML) techniques have shown great promise in predicting properties by fitting high-dimensional nonlinear spaces using large data sets.^10,11^ Recent advancements in property prediction,^12^ preformulation studies,^13^ and drug formulation development^14−16^ highlight the potential of ML-based Quantitative Structure–Property Relationship (QSPR) studies for high-throughput and accurate BCS classification. Ideally, direct prediction of the BCS four classes would be the goal. However, the unavailability of high-quality data sets, due to factors such as data heterogeneity, inconsistency in experimental measurements, and limited access to proprietary data, has led to most studies focusing on the prediction of key properties as an indirect approach for estimating provisional BCS classification. Table 1 summarizes recent advancements in drug BCS prediction, with most employing classification algorithms based on in-house cutoff values for solubility and permeability classification. For these classification processes, data sets (e.g., log *P*_app_ and log *P*) are preclassified using cutoff values, with subsequent model training and testing based on manual-labeled data. Such approach raises two concerns: inappropriate cutoff values can lead to inaccurate data labels, causing significant model bias, and binary classification (high/low) limits the information available for decision-making. A recent study proposed a regression random forest model for permeability classification,^17^ but it was limited to permeability classification, and the number of external validation sets used to assess the actual performance on BCS classification was highly limited, which potentially affects the models’ credibility. Thus, further improvement in ML-driven BCS classification is required. Furthermore, most ML applications for BCS classification rely on traditional ML approaches, and both training and testing data sets are small. This, to a certain extent, restricts the potential for developing more refined predictive models, underscoring the necessity for integrating more comprehensive data sets and adopting advanced methodologies in forthcoming research endeavors.^18^

**Table 1:** Comparison of the Present and Previous Studies on Machine Learning-Based BCS Classification

year	data set	method	task	extra test set
2015^18^	log *S*: 750	decision tree	classification	127 drugs
	log *P*_app_: 1288			
2013^6^	322 oral drugs	linear discriminant analysis; logistic regression; quadratic discriminant analysis	classification	57 drugs
2018^8^	43 oral drugs with log *S* and *P*_eff_	a majority voting system	classification	186 drugs
2022^17^	log *P*_app_: 4462	regression random forest	regression	22 drugs
this work	log *S*: 14,594	XGBoost; LightGBM; Graph Convolutional Networks; Attentive FP; SchNet; ComENet	regression	43 + 294 drugs^a^
	log P: 14,176			
	log *D*: 4101			
	log *P*_app_: 1896			

a 43 drugs for permeability validation, 294 drugs for BCS category validation.

In recent years, advanced ML algorithms have made significant strides. Algorithms like LightGBM and XGBoost have demonstrated impressive results in QSPR studies,^19,20^ while Graph Neural Networks have achieved state-of-the-art performance in many molecular property prediction tasks.^19,21,22^ Another key factor contributing to the success of these ML algorithms is the development of efficient molecular representation methods. These methods effectively encode chemical structures into numerical formats suitable for ML processing. Molecular representations such as molecular fingerprints, graph-based representations, and molecular conformation have been employed in recent studies,^23^ enabling the extraction of relevant information from chemical structures crucial for accurate property prediction and BCS classification. The increasing size and quality of data sets also play a vital role in advancing ML applications for drug property prediction. The growing interest in ML has driven continuous improvement and expansion of BCS-related data sets. Large-scale, high-quality data sets are crucial for training more robust and generalizable models, which can ultimately lead to more reliable BCS classification predictions. Moreover, the development of user-friendly platforms and software tools for ML-based drug property prediction has made advanced techniques more accessible to researchers in drug development.^12−14^ These platforms allow researchers to apply state-of-the-art algorithms to their specific problems without requiring extensive expertise in ML or programming, which facilitates the broader adoption of advanced ML applications in drug development.

To leverage advances in machine learning for BCS classification, we aimed to create a user-friendly, ML-driven web platform for rapid, high-performance BCS class prediction. To achieve this, we first collected substantial data sets on four BCS-related molecular properties: log *S*, log *P*, log *D*, and log *P*_app_. Using these data sets, we developed six machine learning regression models with three different molecular representations to accurately predict each BCS-related property. After that, the best-performing models are used to perform BCS classifications on a data set of marketed oral drugs and human jejunal permeability data to further verify model performance. Lastly, a user-friendly web platform named FormulationBCS was constructed to realize rapid, precise, and end-to-end BCS prediction by simply inputting the structure of query molecules, which aids in the high-throughput assessment of drug candidates at the preformulation stage and shows the potential to streamline the drug approval process.

## 2 Methods

### 2.1 Data Sets

Four data sets, including log *S*, log *D*, log *P*, and log *P*_app_, were collected from various data sources, as summarized in Table 2. For the aqueous solubility data set, 14,594 molecules annotated with experimentally derived log *S* value were collected from the Aquasol data set^24^ and data set made available by Cui et al.^25^ A total of 14,176 molecules with log *P* value were provided by OpenChem,^26^ mainly derived from PHYSPROP.^27^ The log *D* data set (4200 molecules) was from MoleculeNet.^28^ Regarding the log *P*_app_ data set, 1896 molecules with a corresponding experimental value of Caco-2 permeability were collected from the literature,^29^ which is primarily derived from the data set made available by Wang et al.,^30^ the most commonly used data set for Caco-2 permeability prediction.

**Table 2:** Data Volume of the Cleaned Datasets

**data sets**	**data volume**	**source**
log *S*	14,594	Aquasol data set,^24^ Cui et al.^25^data set
log *P*	14,176	OpenChem,^26^ PHYSPROP^27^
log *D*	4101	MoleculeNet^28^
log *P*_app_	1896	Caco-2 permeability literature,^29^ Wang et al.^30^ data set

Data quality is a fundamental issue in machine learning, which can significantly affect the model performance of QSPR tasks. To improve the quality and reliability of data, the process of data cleaning follows four steps:

1. Delete molecules that do not have labels or a simplified molecular input line entry system (SMILES).
2. Canonical SMILES generated with RDKit^31^ was used to identify duplicate entries. After this, if the labels of duplicate entries were not significant different, we took their arithmetic mean as the final label; otherwise, we remove these duplicate items.
3. If a molecule does not contain any carbon atoms, it would be identified as inorganic and be removed.
4. Molecules for which corresponding molecule representation could not be successfully created were removed.

### 2.2 Molecular Featurization

To effectively predict BCS-related properties using machine learning models, it is crucial to ensure that the models comprehensively learn molecular structures. In ML-based QSPR tasks, molecular representations typically stem from three main dimensions: molecule fingerprints and descriptors, molecular graphs, and 3D-atomic coordinates. In this study, we generated these three molecular representations from the molecular structure to serve as inputs for our models.

For molecule fingerprint, Extended-Connectivity Fingerprints (ECFPs) were employed to delineate the structural details of compounds.^32^ ECFPs, a prevalent type of molecular fingerprint in computational chemistry, utilize circular substructures of varying sizes to represent specific groups of atoms within a molecule. These substructures are then hashed into a fixed-length numerical vector comprising 0s and 1s. Molecular descriptors succinctly summarize the structural characteristics of a molecule as well as the physicochemical and electronic features derived or calculated from the structure. They serve as a vital link between a molecule’s structure and its biological activity or other properties, holding a pivotal role in QSPR studies. Incorporating these descriptors into fingerprint representations has been proven to significantly bolster model robustness and enhance performance, as evidenced by numerous studies in computational chemistry.^19,33^ In this study, both ECFPs and molecular descriptors were generated using RDKit,^31^ with the ECFPs characterized by a length and radius of 1024 and 3, respectively.

Molecule graphs are another paradigm of molecular representation. In a graph-based molecular representation, nodes in the graph represent the atoms within the molecule, while the edges represent the chemical bonds connecting these atoms. Node vectors encapsulate various atomic attributes, while edge vectors encompass diverse bond features.^34^ Following Xiong’s work,^22^ we incorporated a comprehensive set of features for both nodes and edges, including nine node features (such as the atom type, atom degree, formal charge, etc.) and four edge features (bond type, conjugation, ring, and stereo). These features were generated by the “AttentiveFPAtomFeaturizer” and “AttentiveFPBondFeaturizer” functions from DGL-LifeSci,^35^ a deep graph library based on Pytorch.^36^

Three-dimensional molecular conformation offers a more intuitive representation, emphasizing the spatial arrangement of atoms within a molecule. This arrangement significantly impacts a molecule’s properties, making it crucial for fields like drug design. In this work, we utilized RDKit to generate and optimize molecular conformations in the MMFF force field.^37^

### 2.3 Machine Learning Model Development

To maximize the information within diverse molecular representations and identify the most effective combination of representations and ML architectures for predicting BCS-related properties, we utilized two representative ML algorithms (LightGBM^38^ and XGBoost^39^) to build the fingerprint and descriptors-based models. Two representative graph-based methods (GCN^21^ and Attentive FP^22^) were used to develop a graph-based model. For capturing molecular conformation information, we applied two representative conformation-based methods (SchNet^40,41^ and ComENet^42^) to develop molecular conformation-based models.

#### 2.3.1 XGBoost (Extreme Gradient Boosting)

XGBoost^39^ (Extreme Gradient Boosting) is an advanced and scalable machine learning framework designed by Tianqi Chen for enhancing decision trees through gradient boosting. This library leverages ensemble learning by integrating multiple simple models to create a more robust and precise model. Central to XGBoost is decision trees that serve as base learners, with the boosting technique fine-tuning their weights to reduce errors. Key features of XGBoost include its parallel processing capabilities for efficiency with large data sets, comprehensive regularization options to prevent overfitting, and its flexibility and interpretability that aid in optimal hyperparameter tuning and understanding feature significance.

#### 2.3.2 Highly Efficient Gradient Boosting Decision Tree (LightGBM)

LightGBM,^38^ another gradient boosting framework based on decision trees, is widely recognized for its efficiency, speed, and scalability in machine learning tasks such as regression and classification. It distinguishes itself through multiple algorithmic optimizations: (1) employing histogram-based binning to minimize data processing and accelerate training; (2) implementing a unique leaf-wise growth strategy with depth restrictions that allows for more precise predictions while mitigating overfitting; and (3) the tree construction in LightGBM selectively includes data points using gradient-based one-side sampling, which improves model accuracy and reduces bias. LightGBM has gained significant attention and optimal performance in property prediction and drug formulation prediction tasks.^20^

#### 2.3.3 Graph Convolutional Networks (GCN)

As of now, various Graph Convolutional Network (GCN) frameworks and variants have been proposed, with the most classical GCN model introduced by Kipf and Welling in their 2017 paper.^21^ The structure of the GCN model is founded on graph convolutions, where each node in the graph is updated through a weighted linear combination of its neighbors’ representations. Specifically, the graph convolutions are defined by eq 1:
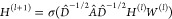
1where *H*(*l*) is the node representation in layer 1; *D* and *A* are the degree matrix and adjacency matrix, respectively; *W*(*l*) is the weight matrix in layer *l*; and σ is the activation function. A distinguishing feature of GCN is its capability to handle graph-structured data, an area where traditional neural network architectures might falter. The primary objective of GCN is to execute node-level prediction tasks, including node classification, link prediction, and clustering on graph-structured data. Its applications span various domains, such as social network analysis, recommendation systems, and bioinformatics.

#### 2.3.4 Attentive FP

Developed by Xiong et al.,^22^ Attentive FP represents a cutting-edge Graph Neural Network (GNN) approach for predicting molecular properties. This model utilizes a recursive neural network (RNN) to progressively gather and update structural information encoded in a molecular graph, moving from local to distant interactions. A distinctive feature of Attentive FP is its incorporation of a graph attention mechanism, which enables the model to selectively concentrate on the most pertinent aspects of the input for an enhanced prediction accuracy. Xiong’s research highlights Attentive FP’s superior performance across a wide range of molecular properties.

#### 2.3.5 SchNet

SchNet is a deep neural network architecture for molecular property prediction, introduced in 2017 by Klaus Schütt.^40,41^ The architecture takes as input a set of 3D coordinates for the atoms in a molecule and predicts various molecular properties such as energies, forces, and dipole moments. The structure of SchNet is based on continuous-filter convolutions, which allow it to capture long-range interactions between atoms in a molecule. A key feature of SchNet is its ability to learn the molecular interactions directly from the input coordinates without relying on hand-engineered molecular features. The goal of SchNet is to achieve high accuracy and efficiency in molecular property prediction, making it a useful tool for numerous applications, particularly in fields such as computational chemistry and materials science.

#### 2.3.6 ComENet

ComENet, proposed by Wang et al.,^42^ is also a cutting-edge graph neural network designed for 3D molecular graph learning. In ComENet, a 3D graph:
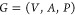
2where *V* is the Node feature matrix, *A* is the adjacency matrix, and *P* is the position matrix. The authors proposed a novel message passing scheme for the complete and efficient processing of 3D information, focusing on both global and local graph details. Notably efficient in computational terms, ComENet excels in handling large data sets and demonstrates superior accuracy in molecular property predictions.

### 2.4 Model Training, Optimization, and Evaluation

For each task, the original data set was divided into three parts: training (80%), validation (10%), and testing subsets (10%), utilizing random stratified splitting techniques. The training subset was used for model training, while the validation subset played a crucial role in tuning the hyperparameters to achieve the optimal configuration. After tuning, the training and validation subsets were combined, and the models were evaluated using a 5-fold cross-validation method on the combined data set to assess their stability and robustness. Finally, the testing subset was employed to assess the final generalization capability of the model. This approach, encompassing model training, validation, and testing, is a widely recognized standard practice in ML. The Tree-structured Parzen Estimator (TPE) algorithm,^43^ implemented using the hyperopt library,^44^ was utilized to identify the optimal hyperparameters for ML models through 50 evaluations (with 30 evaluations for predictions on log *S* and log *P* by the SchNet and ComENet models due to their high computing overhead). Hyperopt is renowned as one of the most used Bayesian optimizers, encompassing a range of optimization algorithms, such as random search and the TPE approach. In comparison to Bayesian optimization methods based on Gaussian processes, TPE, utilizing Gaussian mixture models, generally delivers superior results with greater efficiency across most scenarios.^43^ This has led to its widespread adoption in Automated Machine Learning (AutoML).^43^ To prevent overfitting and excessive time consumption, all Neutral Network (NN)-based models were trained with early stopping after 50 epochs, halting training when no improvement in the validation performance was observed. For deep learning models, we performed additional manual fine-tuning of their hyperparameters. This was necessary as the hyperparameter search space of DNNs is extensive, and direct employment of Bayesian search may lead to underfitting or overfitting compared with other learning algorithms. Model evaluation primarily focuses on three key metrics: the coefficient of determination (*R*^2^), Mean Absolute Error (MAE), and Root Mean Squared Error (RMSE).

### 2.5 External Validation Data

#### 2.5.1 BCS Category Data of Marketed Drugs

**Figure 2:**
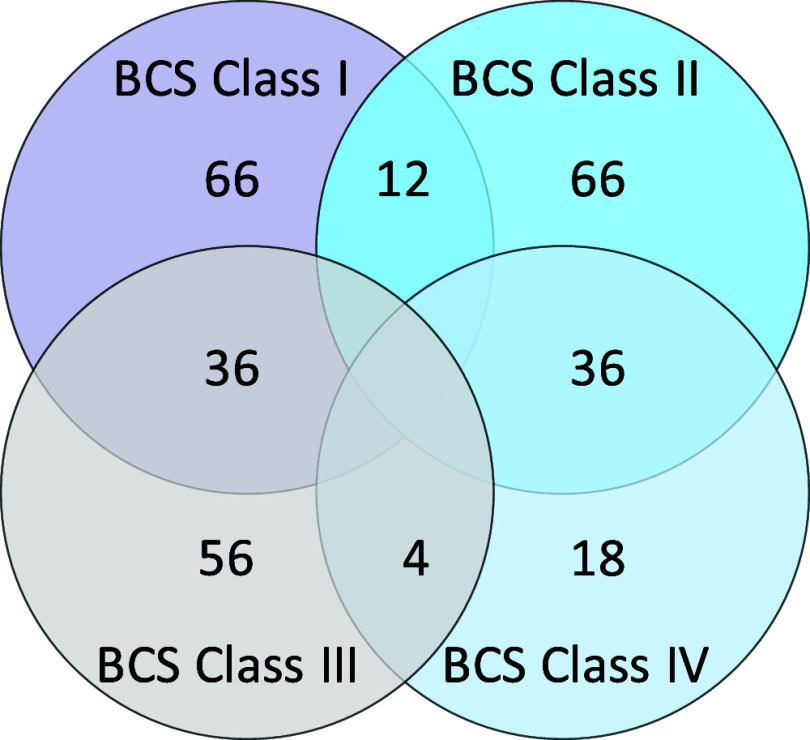
BCS category distribution among the collected marketed drug data set.

To further validate the effectiveness of the BCS-related discriminant models, we assembled marketed drug BCS data from the FDA^45^ and WHO^46^ reports and publicly available literatures^3,47−49^ as the external test set. The raw data underwent deduplication, and conflicting entries on solubility or permeability from different sources were removed. After data cleaning, a total of 294 marketed drug data was collected (Supporting Information), including 66 BCS class 1 drugs, 66 BCS class 2 drugs, 56 BCS class 3 drugs, 18 BCS class 4 drugs, 36 BCS class 1/3 drugs (high solubility), 36 BCS class 2/4 drugs (low solubility), 12 BCS class 1/2 drugs (high permeability), and 4 BCS class 3/4 drugs (low permeability), as shown in Figure 2.

#### 2.5.2 Human Jejunal Permeability Data

Given that permeability values obtained from *in vivo* human studies are the most reliable and realistic data for drug permeability validation, we employed the data on 43 compounds with known human jejunal permeability values^8^ to further validate our permeability models.

### 2.6 Web Platform Construction

To meet the industry’s demand for a high-efficiency, stable, and scalable platform, we developed an online, agile, and expandable platform for streamlined BCS classification. The system is built on Alibaba Cloud’s Elastic Compute Service (ECS) for essential hardware infrastructure, with Ubuntu as the server’s operating system. We utilized uWSGI and Nginx for efficient and secure web request management, load balancing, and enhanced security. Python was chosen as the primary programming language due to its popularity and robust ecosystem, including AI and data processing packages such as Numpy, Pandas, Scikit-learn, and PyTorch. We employed Django as the framework to ensure a clear separation of business data (Model), user interfaces (Template), and business logic (View), facilitating easier upgrades and maintenance. MySQL was used for data storage due to its reliability as a relational database engine. For the front-end, we used CSS and JavaScript to create a cross-platform user interface and AJAX to connect the front-end with the back-end. We implemented the Transport Layer Security (TLS) protocol for data transmission. This comprehensive architecture enabled our BCS prediction platform to efficiently store and format evaluated models, providing users with reliable BCS classification prediction services.

## 3 Results and Discussions

### 3.1 Model Performance

**Figure 3:**
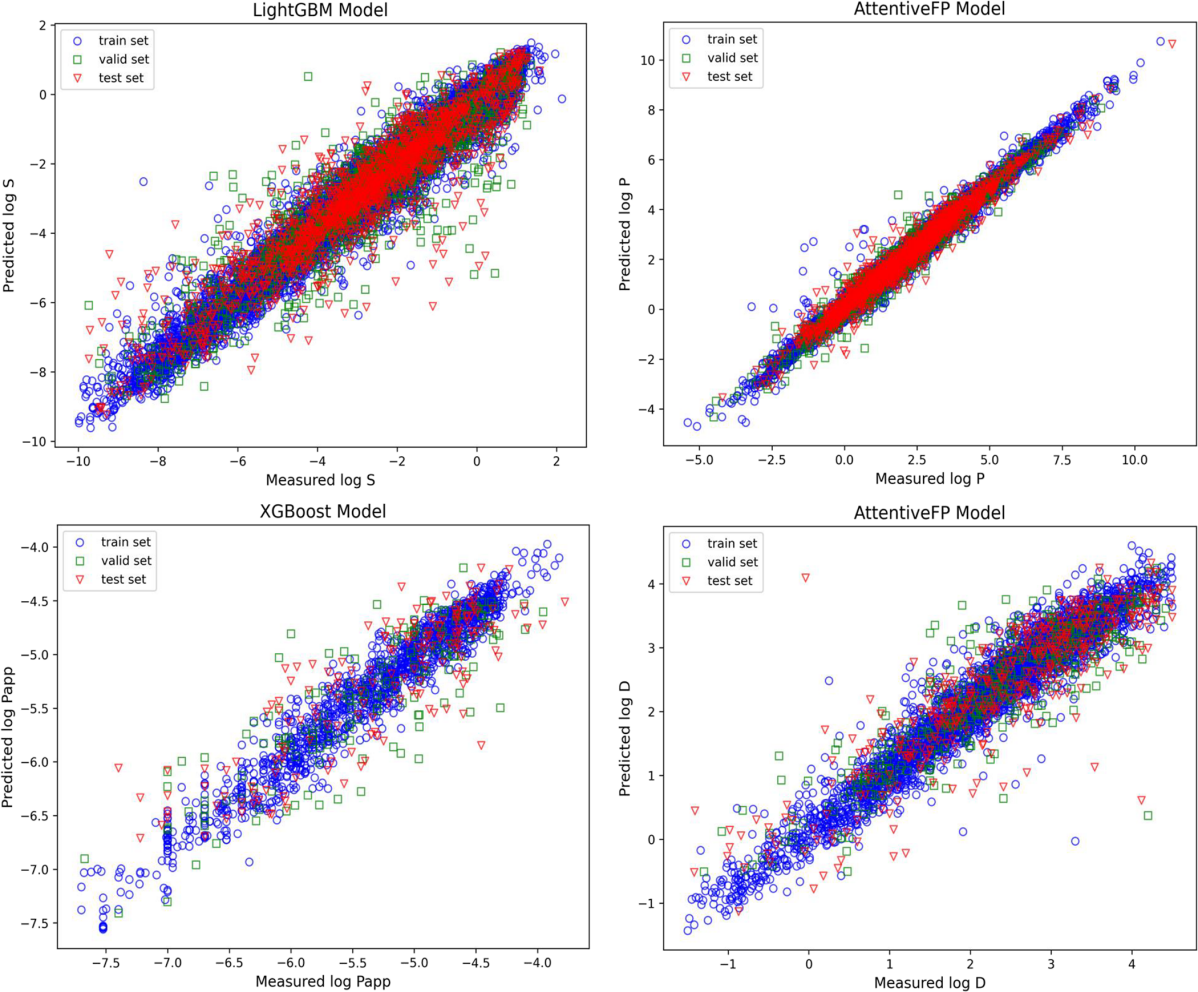
Scatter plots of the best model predictions for the four tasks. The horizontal axis represents the measured values, and the vertical axis represents the predicted values. Blue circles denote training data, green squares denote validation data, and red triangles denote test data. Upper left: log *S* prediction with LightGBM, upper right: log *P* prediction with Attentive FP, lower left: log *P*_app_ prediction with XGBoost, lower right: log *D* prediction with Attentive FP.

Table 3 shows the performance of six machine learning methods (XGBoost, lightGBM, AttentiveFP, GCN, SchNet, and ComENet) for four tasks (log *S*, log *D*, log *P*, and log *P*_app_). Table 4 presents the performance and standard deviations from the 5-fold cross validation of six machine learning methods across four tasks. The cross-validation results indicate that the models exhibit stable performance across both the training and validation sets, with small standard deviations showing that model performance is not significantly affected by specific data splits. Figure 3 shows the scatterplot of the optimal model for each task. For solubility prediction, the *R*^2^ values were around 0.8 across the models. SchNet had the lowest accuracy at 0.74, while LightGBM had the highest at 0.84. This trend was consistent for both RMSE and MAE, with SchNet recording the lowest accuracies of 1.12 and 0.76, respectively, and LightGBM achieving the highest accuracies of 0.88 and 0.59. Among the four models based on raw molecular structure information (2D graph and 3D conformation), the AttentiveFP model showed superior performance, nearing the accuracy of the descriptor-based model. This indicates that AttentiveFP effectively captures molecular feature information using only a limited set of atomic and bond properties. For permeability related tasks, two graph-based methods and two fingerprint and descriptors-based methods achieve comparable performances with the RMSE of 0.40 for the test set in log *P* prediction. For log *D* prediction, AttentiveFP outperformed others, achieving the lowest RMSE of 0.60 in the test set, while GCN had a slightly higher RMSE of 0.62. The performances of two descriptors-based methods and two conformation-based methods are obviously unpleasant on this data set. Regarding log *P*_app_ prediction, XGBoost performs best with the RMSE and *R*^2^ of 0.42 and 0.71 for the test set, respectively, slightly surpassing the results of LightGBM, GCN, and AttentiveFP. The quality of the data set directly determines the upper limit of the model’s predictive performance. It has been reported that the standard deviation of experimental solubility values for the same compound can be as high as 0.5 in logS units.^24^ The experimental error in measuring log *P* ranges from 0.2 to 0.4 log units,^51^ while for log *D*, this value falls between 0.11 and 0.27.^52^ The experimental error for log *P*_app_ measurements is between 0.3 and 0.7 log units.^53^ Experimental errors constitute the main source of data errors. In future work, improving the methods for determining BCS properties and reducing experimental errors will benefit the models.

**Table 3:** Model Performance on the Training Set, Validation Set, and Test Set for Four Tasks

		training set			validation set				test set	
property	model	RMSE	MAE	*R*^2^	RMSE	MAE	*R*^2^	RMSE	MAE	*R*^2^
log *S*	LightGBM	0.49	0.35	0.95	0.88	0.61	0.84	**0.88**	**0.59**	**0.84**
	XGBoost	0.32	0.23	0.98	0.88	0.60	0.84	0.89	0.60	0.84
	GCN	0.75	0.55	0.88	0.92	0.65	0.82	0.98	0.66	0.81
	AttenFP	0.82	0.58	0.86	0.90	0.64	0.83	0.89	0.62	0.84
	SchNet	0.64	0.46	0.91	0.95	0.70	0.80	1.16	0.80	0.74
	ComENet	0.66	0.47	0.90	0.90	0.66	0.81	1.12	0.76	0.76
log *P*	LightGBM	0.23	0.16	0.98	0.43	0.29	0.94	0.42	0.30	0.95
	XGBoost	0.15	0.10	0.99	0.43	0.30	0.94	0.42	0.30	0.95
	GCN	0.21	0.15	0.98	0.37	0.26	0.96	0.39	0.27	0.95
	AttenFP	0.24	0.17	0.98	0.34	0.24	0.96	**0.36**	**0.25**	**0.96**
	SchNet	0.39	0.30	0.95	0.51	0.35	0.92	0.55	0.39	0.91
	ComENet	0.31	0.24	0.96	0.44	0.27	0.94	0.48	0.32	0.94
log *D*	LightGBM	0.18	0.16	0.97	0.67	0.50	0.66	0.71	0.54	0.66
	XGBoost	0.27	0.21	0.95	0.71	0.55	0.62	0.72	0.56	0.65
	GCN	0.32	0.24	0.93	0.56	0.42	0.76	0.62	0.47	0.73
	AttenFP	0.32	0.24	0.93	0.55	0.40	0.78	**0.60**	**0.43**	**0.76**
	SchNet	0.57	0.43	0.77	0.61	0.48	0.72	0.71	0.54	0.66
	ComENet	0.45	0.36	0.82	0.58	0.45	0.74	0.63	0.47	0.72
log *P*_app_	LightGBM	0.15	0.12	0.96	0.38	0.30	0.74	0.42	0.33	0.70
	XGBoost	0.17	0.13	0.95	0.40	0.31	0.72	**0.42**	**0.33**	**0.71**
	GCN	0.31	0.24	0.84	0.42	0.33	0.69	0.42	0.34	0.70
	AttenFP	0.30	0.23	0.85	0.39	0.30	0.73	0.43	0.33	0.69
	SchNet	0.40	0.33	0.75	0.42	0.34	0.69	0.48	0.38	0.64
	ComENet	0.37	0.29	0.78	0.41	0.32	0.71	0.45	0.35	0.68

**Table 4:** Five-Folds Cross-Validation Results for Four Tasks

		training set in 5-folds			validation set in 5-folds		
property	model	RMSE	MAE	*R*^2^	RMSE	MAE	*R*^2^
log *S*	LightGBM	0.475(0.004)^a^	0.339(0.003)	0.953(0.001)	0.920(0.040)	0.624(0.019)	0.824(0.015)
	XGBoost	0.442(0.004)	0.323(0.003)	0.960(0.002)	0.895(0.045)	0.623(0.024)	0.828(0.020)
	GCN	0.685(0.020)	0.487(0.021)	0.904(0.009)	0.973(0.027)	0.646(0.012)	0.804(0.014)
	AttenFP	0.714(0.023)	0.507(0.026)	0.894(0.012)	0.953(0.023)	0.640(0.011)	0.811(0.010)
	SchNet	0.629(0.032)	0.434(0.036)	0.925(0.021)	1.021(0.072)	0.684(0.047)	0.781(0.035)
	ComENet	0.667(0.032)	0.465(0.035)	0.912(0.018)	0.989(0.056)	0.654(0.038)	0.801(0.029)
log *P*	LightGBM	0.221(0.002)	0.154(0.001)	0.985(0.001)	0.464(0.013)	0.311(0.003)	0.935(0.004)
	XGBoost	0.134(0.002)	0.088(0.001)	0.994(0.001)	0.461(0.016)	0.305(0.006)	0.936(0.004)
	GCN	0.236(0.017)	0.162(0.012)	0.982(0.003)	0.422(0.011)	0.264(0.005)	0.948(0.003)
	AttenFP	0.224(0.013)	0.155(0.009)	0.985(0.002)	0.396(0.007)	0.252(0.003)	0.953(0.002)
	SchNet	0.415(0.025)	0.331(0.021)	0.946(0.012)	0.527(0.029)	0.385(0.012)	0.915(0.008)
	ComENet	0.305(0.025)	0.236(0.018)	0.963(0.011)	0.491(0.025)	0.366(0.010)	0.921(0.008)
log *D*	LightGBM	0.303(0.003)	0.228(0.003)	0.936(0.001)	0.654(0.030)	0.491(0.026)	0.700(0.024)
	XGBoost	0.313(0.001)	0.234(0.002)	0.931(0.001)	0.654(0.026)	0.487(0.021)	0.700(0.019)
	GCN	0.348(0.039)	0.267(0.027)	0.910(0.017)	0.575(0.020)	0.416(0.016)	0.761(0.018)
	AttenFP	0.296(0.037)	0.223(0.027)	0.937(0.016)	0.552(0.016)	0.396(0.013)	0.786(0.016)
	SchNet	0.583(0.048)	0.426(0.035)	0.782(0.020)	0.626(0.032)	0.461(0.026)	0.715(0.025)
	ComENet	0.518(0.035)	0.389(0.026)	0.810(0.016)	0.605(0.023)	0.444(0.015)	0.738(0.018)
log *P*_app_	LightGBM	0.156(0.002)	0.121(0.002)	0.960(0.002)	0.399(0.020)	0.308(0.015)	0.732(0.028)
	XGBoost	0.181(0.002)	0.139(0.001)	0.945(0.001)	0.394(0.016)	0.306(0.013)	0.739(0.019)
	GCN	0.351(0.026)	0.274(0.020)	0.812(0.025)	0.414(0.029)	0.325(0.022)	0.692(0.047)
	AttenFP	0.314(0.022)	0.258(0.018)	0.838(0.024)	0.398(0.025)	0.306(0.018)	0.736(0.032)
	SchNet	0.416(0.034)	0.314(0.028)	0.733(0.032)	0.441(0.033)	0.343(0.029)	0.668(0.047)
	ComENet	0.376(0.031)	0.295(0.027)	0.763(0.029)	0.432(0.030)	0.332(0.025)	0.685(0.045)

a The results in the table are presented in the form of “mean (standard deviation)”, and all experimental results are obtained through 5-fold cross validation.

Overall, as shown in Table 3, LightGBM, XGBoost, and Attentive FP generally perform better than the other models, while graph methods based on 3D coordinates perform worst on all tasks. This is contrary to common sense, as the 3D structure of molecules is crucial for their properties and drug actions.^54^ Similar phenomenon is also noted in several studies,^55,56^ which can be attributed to three factors. First, computation constraints limit the hyperparameter optimization for these models. Second, graph networks based on 3D coordinates are designed for quantum interactions and need large data sets with quantum mechanics characteristics, resulting in poor performance on smaller data sets. These models also ignore predefined bonding information, necessitating a large data corpus to discern atomic relationships, such as bonded or nonbonded states.^57^ Third, these models require accurate spatial information. They extend spatial data to complex spaces, making them very sensitive to the accuracy of three-dimensional information. However, in this work, the 3D structures rapidly generated by RDKit in a vacuum state are approximated and noisy, differing from the conformations of molecules in solvents or even in physiological conditions. In future work, it may be possible to improve model performance by introducing solvent environments or using multiconformation sampling techniques.

Regarding lipophilicity prediction, models based on fingerprints and descriptors perform lower for log *D* compared to the other two types of models, contrasting with the overall prediction results. This indicates that the current combination of molecular fingerprints and descriptors lacks crucial variables for log *D*. Predicting log *D* is more complex than log *P*, as it requires understanding the molecule’s ionization state at a given pH (typically pH 7.4 in drug development).^58,59^ In fact, the pH dependent distribution coefficient, log *D*, is related to log *P* through the ionization constant, p*K*_a_. Log *D* can be derived from log *P* and p*K*_a_ for a singly ionized substance at a given pH with eqs 3 and 4 for acids and bases, respectively:^60^
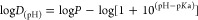
3
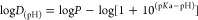
4

This also highlights that log *P* is a critical parameter in descriptor-based log *D* prediction. To validate this, we added the predicted log *P* variable and retrained the LightGBM model for log *D* prediction, resulting in a notable improvement in model performance with an RMSE of 0.64. This result suggests that feature engineering is necessary to achieve good performance, especially in descriptor-based models, and further indicates that GNN models may be more competitive when there is no prior knowledge of choosing more appropriate descriptors.

### 3.2 External Validation

#### 3.2.1 Solubility Classification Performance

The classification of high and low solubilities for the 278 compounds with clear solubility labels was determined using the dose number (*D*_0_), which can be derived from the following equation:^7^
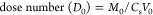
5where *M*_0_ (in milligrams) is the highest single therapeutic dose that was manually collected from the drug product labels, *C*_s_ (in milligrams per milliliter) is the solubility value predicted with our current optimal solubility model, and *V*_0_ is set at 250 mL.

The solubility classification results based on the calculated *D*_0_ is shown in Table 5. *D*_0_ greater than 1 implies low solubility, while *D*_0_ less than or equal to 1 implies high solubility. The prediction result demonstrated excellent consistency with the referenced BCS labels, achieving a total accuracy of 77.7%. The performance on the external validation set is particularly strong for low solubility drugs, with an accuracy of around 85.8%, and satisfactory for BCS Class 1 and 3 drugs, with an accuracy of approximately 71.5%. It is notable that determining drug solubility based on the highest single therapeutic dose might limit the application of BCS classification, as the dose value of drugs is typically evaluated in the latter stages of drug development.^4^ To overcome this constraint, we also used the lower limit of the solubility range defined in the USP (0.1 mg/mL) as the cutoff value. It was observed that using this cutoff value also yielded acceptable results for the external test data, with an accuracy of 73%.

**Table 5:** Confusion Matrix in Solubility Classification^a^

log *S*	predicted high solubility	predicted low solubility	total	accuracy (%)
high solubility	113	45	158	71.5
low solubility	17	103	120	85.8
total	130	148		
precision (%)	**86.9**	**69.6**		

a Total accuracy: (113 + 103) / (113 + 45 + 17 + 103) = 77.7%.

#### 3.2.2 Permeability Classification Performance

Here, we compare the external validation of permeability classification based on the optimal models for three tasks: log *P*, log *D*, and log *P*_app_. The boundaries for classifying permeability were established by setting a cutoff value based on the benchmark provided by the internal standard drug, Metoprolol. For log *P*, the boundary is generally confirmed as 1.72.^61^ Due to variations in the methodology and conditions used for estimating log *D*, there is no universally accepted exact value for log *D* of Metoprolol at pH 7.4. In this study, we used the predicted log *D* value of Metoprolol (−0.1954) with the AttentiveFP model as the cutoff value. Regarding log *P*_app_, although Metoprolol reports a permeability value of log *P*_app_ 4.7 (*P*_app_ = 20 × 10^–6 cm/s), many drugs with lower *P*_app_ values than metoprolol are often considered to be fully absorbed. This is because Metoprolol’s Fa value (Fa ≥ 95%) is more conservative than the standards of FDA and EMA (Fa ≥ 85%).^7^ Based on previous studies,^62,63^ a *P*_app_ value of 8.0 × 10^–6 cm/s (log *P*_app_= −5.097) is employed as the Caco-2 permeability cutoff value, which has been used for identifying compounds with Fa ≥ 85%.

With the corresponding cutoff values, we applied the three optimal models to predict the permeability of 222 drugs with permeability labels from their provisional BCS classes. The performance of three models are depicted in Tables 6–8, respectively, indicating that these models exhibited acceptable performance in permeability classification, with accuracy ranging from 71.2 to 73.4%. Among them, the log *P*_app_ model was found to be the most informative predictor for drug permeability, achieving the highest accuracy of 73.4%, and both sensitivity and specificity were close to 73%, which showcases that the log *P*_app_ prediction model possesses a balanced ability to correctly classify drugs as low or high permeability. Although the models of log *D* and log *P* also showed comparable overall performance, both models exhibit bias toward a certain label. Specifically, log *P* has an accuracy of 75.6% for low permeability drugs, but only 68.6% for high permeability; log *D*, on the other hand, performs better in predicting high permeability drugs with a sensitivity of 84%, but has a specificity of less than 50%, making it ineffective in determining low permeability drugs. The reason for this might be that, from an ML modeling perspective, the log *D* model has the largest MAE value of 0.43 compared to log *P* and log *P*_app_. When using the predicted log *D* value (−0.19) as the cutoff, more than 20 out of 79 drugs labeled as low permeability fall within the range of (−0.19 – MAE, −0.19 + MAE), indicating high uncertainty in the classification of low permeability drugs when the cutoff fluctuates slightly. From a biophysical perspective, both log *D* and log *P* indicate the passive diffusion permeability of drugs across the intestinal wall. However, they fall short in classifying the permeability of drugs that are actively absorbed via transporters. Carrier-mediated absorption relies on specific drug–protein interactions, which are distinct from the processes governed by lipophilicity.

**Table 6:** Permeability Classification Confusion Matrix Based on log *P*^a^

log *P*	predicted high permeability	predicted low permeability	total	accuracy (%)
high permeability	99	45	144	68.8
low permeability	19	59	78	75.6
total	118	104		
precision (%)	**83.9**	**56.7**		

a Total accuracy: (99 + 59) / 222 = 71.2%.

**Table 7:** Permeability Classification Confusion Matrix Based on log *D*^a^

log *D*	predicted high permeability	predicted low permeability	total	accuracy (%)
high permeability	123	21	144	85.4
low permeability	40	38	78	48.7
total	163	59		
precision (%)	**75.5**	**64.4**		

a Total accuracy: (123 + 38) / 222 = 72.5%.

**Table 8:** Permeability Classification Confusion Matrix Based on log *P*_app_^a^

log *P*_app_	predicted high permeability	predicted low permeability	total	accuracy (%)
high permeability	105	39	144	73.0
low permeability	20	58	78	74.3
total	125	97		
precision (%)	**84**	**60**		

a Total accuracy: (105 + 58) / 222 = 73.4%.

Apart from validating with marketed drugs with provisional BCS labels, we employ the human jejunal permeability data set to access permeability classification performance. As delineated in Table 9, the performance varies significantly. Specifically, the Caco-2-derived log *P*_app_ prediction model achieves a superior predictive accuracy at 74.4% for the group of 43 compounds, compared to 55.8 and 58.1% for the log *P* and log *D* prediction models, respectively. However, using the log *P*_app_ model for drug permeability prediction still has limitations. While Caco-2 monolayers can predict passive drug transport and provide insights for carrier-mediated systems, variations in carrier expression and differences from *in vivo* conditions may lead to discrepancies,^64^ particularly with actively transported drugs like l-leucine, l-dopa, and d-glucose in the human jejunal permeability test set. Additionally, the high experimental cost results in a smaller log *P*_app_ data set compared to lipophilicity data sets, potentially limiting model generalizability. Therefore, in the long run, collecting more high-quality Caco-2 data is an essential task. Expanding and diversifying the data set will enhance the model’s predictive performance and overall reliability.

**Table 9:** External Validation Result for Permeability Prediction with the Human Jejunal Permeability Dataset^8^

**drug**	**BCS class**	Pred_log *P* (cutoff: 1.720)	Pred_log *D* (cutoff: −0.195)	**Pred_log *P***_**app**_**(cutoff: −5.097)**
acetaminophen	1	0.523	**0.406**	**–4**.**539**
amiloride hydrochloride	1	0.756	–0.372	–6.001
amoxicillin trihydrate	3	**–0.286**	**–1.320**	**–6**.**340**
antipyrine	1	0.588	**0.156**	**–4**.**180**
atenolol	3	**0.315**	**–0.987**	–5.286
benserazide	1	–2.197	–0.198	–6.586
varbamazepine	2	**2.313**	**1.881**	**–4**.**533**
vephalexin	1	0.395	–1.030	–5.870
vimetidine	3	**0.627**	**–0.688**	**–5**.**797**
vreatinine	3	**–1.430**	**–1.288**	**–5**.**376**
vyclosporine	2	**2.400**	**3.451**	–5.822
d-glucose	1	–2.977	–2.276	–6.002
desipramine	1	**4.216**	**1.196**	**–4**.**840**
enalapril maleate	1	1.040	**–0.082**	–5.357
enalaprilat	3	**–0**.**912**	**–1.177**	**–6**.**120**
fexofenadine	3	3.078	0.427	**–5**.**190**
fluvastatin	1	**3.890**	**1.705**	–5.537
furosemide	4	2.070	**–0.890**	**–5**.**745**
griseofulvin	2	**2.254**	**2.130**	**–4**.**300**
hydrochlorothiazide	3	**–0.172**	–0.008	**–5**.**971**
hydrocortisone	1	1.482	**0.489**	**–5**.**030**
isotretinoin	2	**6.240**	**3.307**	**–4**.**384**
inogatran	3	**–0.156**	**–0.506**	**–6**.**120**
ketoprofen	2	**3.261**	**0.023**	**–4**.**586**
l-leucine	1	–1.624	–1.095	–5.167
l-dopa	1	–2.547	–1.006	–5.820
lisinopril	3	**–1.850**	**–1.060**	**–6**.**426**
losartan	3	3.437	3.701	**–5**.**620**
methyldopa	3	**–2.359**	0.194	**–5**.**500**
metoprolol	1	**1.821**	–0.195	**–4**.**809**
naproxen	2	**3.071**	**–0**.**079**	**–4**.**535**
phenylalanine	1	–1.412	–1.005	**–5**.**145**
piroxicam	2	**2.852**	–0.242	**–4**.**850**
propranolol	1	**3.152**	**0.827**	**–4**.**651**
ranitidine	3	**0.023**	**–0.930**	**–5**.**885**
salicylic acid	1	1.523	–0.922	**–4**.**779**
sulforaphane	2	0.725	–0.619	**–4**.**618**
talinolol	3	2.898	1.597	**–5**.**237**
terbutaline	3	**0.512**	**–0.382**	**–5**.**527**
triamcinolone acetonide	2	**2.329**	**1.653**	**–4**.**988**
urea	1	–1.781	–0.748	**–4**.**992**
valacyclovir	1	–0.662	–1.059	–5.940
verapamil hydrochloride	1	**3.868**	**2.907**	**–5**.**010**
**accuracy**		**55.8%**	**58.1%**	**74.4%**

#### 3.2.3 Performance of BCS Classification

Following the solubility and permeability classification performance evaluation, the BCS classification was evaluated on 206 compounds with unique BCS labels using the LightGBM model for solubility prediction and the XGBoost model on log *P*_app_ for permeability determination. As observed in Table 10, more than half of the data (54.8%) were classified correctly. About 40% of the data were partially classified correctly (either solubility or permeability), while only eight compounds (3.9%) were entirely misclassified. The results indicate that the established machine learning models demonstrate a reasonable capability for predicting the BCS classification of drugs based on solubility and permeability.

**Table 10:** Performance for BCS Class Prediction

**BCS prediction**		**BCS class**			
		BCS 1	BCS 2	BCS 3	BCS 4
**predicted BCS class**	BCS 1	**33**	8	12	0
	BCS 2	19	**39**	3	1
	BCS 3	11	2	**31**	7
	BCS 4	3	17	10	**10**

### 3.3 Web Platform for BCS Classification

**Figure 4:**
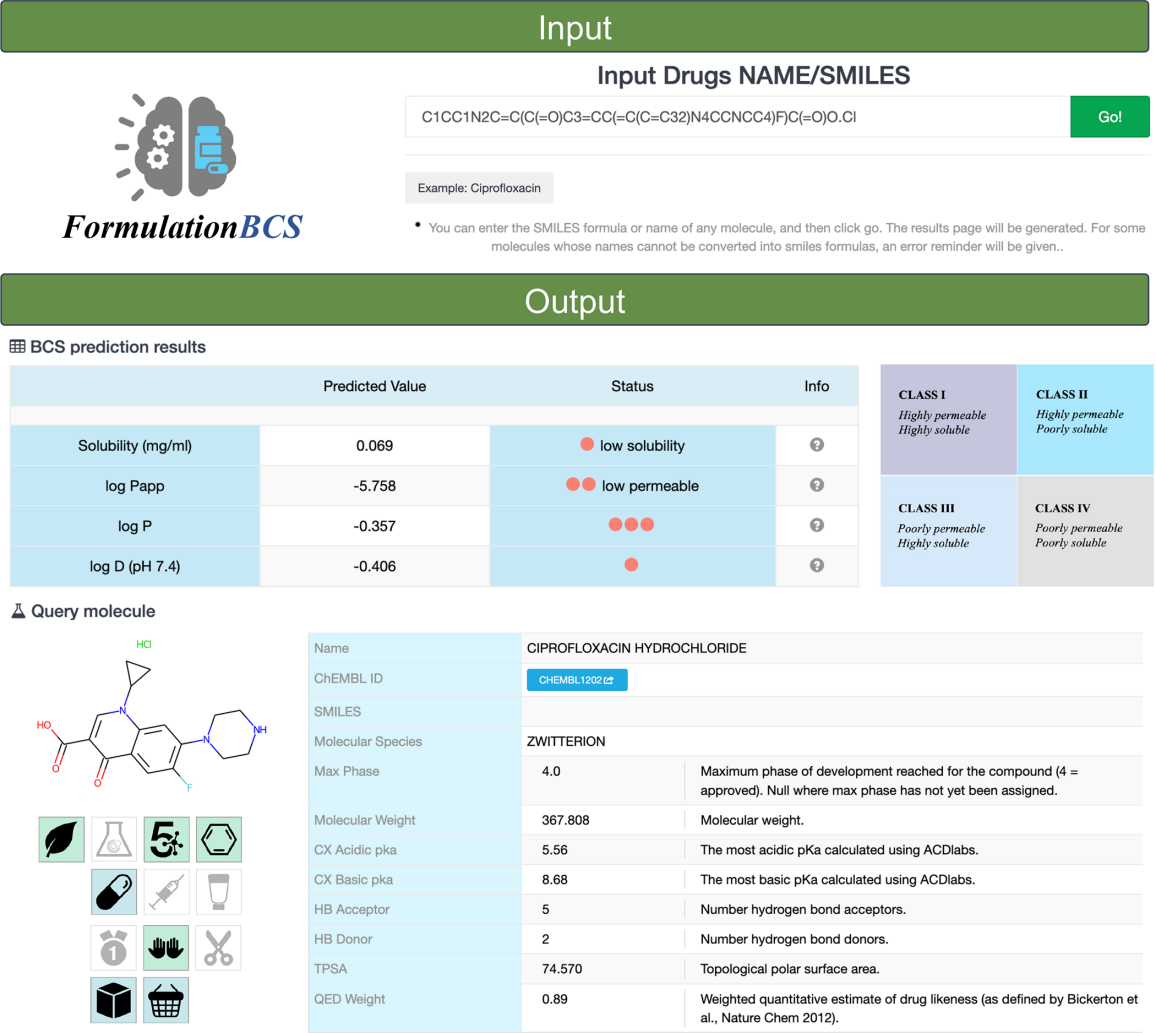
Snapshot of the user interface and application overview of FormulationBCS.

To reduce the barriers to using machine learning models and expand their application scenarios, we have deployed the optimized model on a user-friendly web platform named FormulationBCS. FormulationBCS is an online platform that supports end-to-end BCS classification prediction, with an overview of its user interface provided in Figure 4. The platform is designed to streamline the prediction process and ensure a smooth user experience through a clear and intuitive interface. Users simply input the SMILES of a molecule or the name of a drug, and FormulationBCS automatically performs subsequent calculations within seconds, outputting a wealth of information, including BCS classification and predictions of quantitative BCS properties such as solubility, log *D*, log *P*, and log *P*_app_. Additionally, key molecular properties, such as molecular weight and polar surface area, will also be computed and provided. All results are presented through intuitive text formats or interactive charts, enhancing their comprehensibility. FormulationBCS is freely accessible at http://formulationbcs.wztgyh.com and requires no additional software or hardware installation, making it easily usable on any device with a web browser. We believe that FormulationBCS will become an indispensable tool in drug development, aiding researchers in making more informed decisions during the early stages of drug discovery.

## 4 Conclusions

In the present work, a high-performance machine learning-based BCS online prediction platform (FormulationBCS) was successfully established. After diverse molecular representations and learning algorithms were compared, descriptor-based models (LightGBM and XGBoost) and a graph-based model (AttentiveFP) demonstrated superior predictive performance in predicting BCS properties. The top-performing models were further validated using the approved drug BCS category data set. Such optimal models were deployed on a user-friendly web platform, enabling an automated end-to-end BCS class prediction. FormulationBCS exhibits satisfactory predictive performance while covering a broad chemical space, providing pharmaceutical researchers with a valuable tool for the high-throughput drug candidate BCS class evaluation. This will further aid in developability assessments and drug development decisions, contributing significantly to efficiency improvement and risk reduction for drug development. In future work, improvements in data scale and quality, the development of multimodal representation methods, and the introduction of transfer learning and multitask learning strategies are expected to further enhance the model’s predictive performance and generalization ability. The FormulationBCS platform will also undergo continuous updates based on user feedback to expand its functionality and improve stability.

## Data Availability

The data sets and models are accessible at https://github.com/NamanWang/FormulationBCS. The FormulationBCS web platform is freely accessible at http://formulationbcs.wztgyh.com/

## Supporting Information Available

External validation data set: BCS category data of 294 marketed drugs (Table S1) (PDF)

## Abbreviations

BCS: biopharmaceutics classification system
ML: machine learning
QSPR: quantitative structure–property relationships
IR: immediate-release
BE: bioequivalence
EMA: European Medicines Agency
FDA: U.S. Food and Drug Administration
CADD: computer-aided drug design
ECFPs: Extended-Connectivity Fingerprints
XGBoost: Extreme Gradient Boosting
LightGBM: Highly Efficient Gradient Boosting Decision Tree
GCN: Graph convolutional networks
GNN: Graph Neural Network
RNN: recursive neural network
TPE: Tree-structured Parzen Estimator
AutoML: Automated Machine Learning
*R*^2^: coefficient of determination
MAE: Mean Absolute Error
RMSE: Root Mean Squared Error
ECS: Elastic Compute Service
CSS: Cascading Style Sheets
AJAX: Asynchronous Javascript And XML
TLS: Transport Layer Security
