# Supplementary material for: FormulationBCS: A Machine Learning Platform Based on Diverse Molecular Representations for Biopharmaceutical Classification System (BCS) Class Prediction
Source: Mol Pharm. 2024 Dec 8;22(1):330–42. doi: 10.1021/acs.molpharmaceut.4c00946 (PMC11707745; doi:10.1021/acs.molpharmaceut.4c00946)
Supplement: Supplementary file 1 — mp4c00946_si_001.pdf [file mp4c00946_si_001.pdf]

Supporting Information

FormulationBCS: a machine learning platform based on diverse molecular representations for Biopharmaceutical Classification System (BCS) class prediction

Zheng Wu[a,1], Nannan Wang[a,1], Zhuyifan Ye[b], Huanle Xu[c], Ging Chan[a,d], Defang Ouyang[a,d, *]

[a] Institute of Chinese Medical Sciences (ICMS), State Key Laboratory of Quality Research in Chinese Medicine, University of Macau, Macau 999078, China

[b] Faculty of Applied Sciences, Macao Polytechnic University, Macau 999078, China

[c] Faculty of Science and Technology, University of Macau, Macau 999078, China

[d] Department of Public Health and Medicinal Administration, Faculty of Health Sciences (FHS), University of Macau, Macau 999078, China

[*] Corresponding author: Defang Ouyang, Email: defangouyang@um.edu.mo

[1] These authors contributed equally to this article

Table S1 The BCS category of 294 marketed drugs

Drug_Name	SMILES	BCS category
Estradiol	[H][C@@]12CC[C@H](O)[C@@]1(C)CC[C@]1([H])C3=C(CC[C@@]21[H])C=C(O)C=C3	1
Atropine	CN1[C@H]2CC[C@@H]1C[C@@H](C2)OC(=O)C(CO)C1=CC=CC=C1	1
Norgestimate	[H][C@@]12CC[C@@](OC(C)=O)(C#C)[C@@]1(CC)CC[C@]1([H])[C@@]3([H])CC\C(C=C3CC[C@@]21[H])=N/O	1
Clonidine Hydrochloride	C1CN=C(N1)NC2=C(C=CC=C2Cl)Cl.Cl	1
Norethindrone	C[C@]12CC[C@H]3[C@H]([C@@H]1CC[C@]2(C#C)O)CCC4=CC(=O)CC[C@H]34	1
Norgestrel	CC[C@]12CC[C@H]3[C@H]([C@@H]1CC[C@]2(C#C)O)CCC4=CC(=O)CC[C@H]34	1
Anastrozole	CC(C)(C#N)C1=CC(=CC(CN2C=NC=N2)=C1)C(C)(C)C#N	1
Indapamide	CC1CC2=CC=CC=C2N1NC(=O)C1=CC(=C(Cl)C=C1)S(N)(=O)=O	1
Alprazolam	CC1=NN=C2CN=C(C3=CC=CC=C3)C3=C(C=CC(Cl)=C3)N12	1
Lorazepam	OC1N=C(C2=CC=CC=C2Cl)C2=C(NC1=O)C=CC(Cl)=C2	1
Chlorpheniramine	CN(C)CCC(C1=CC=C(Cl)C=C1)C1=CC=CC=N1	1
Amiloride	C1(=C(N=C(C(=N1)Cl)N)N)C(=O)N=C(N)N	1
Desloratadine	ClC1=CC2=C(C=C1)C(=C1CCNCC1)C1=C(CC2)C=CC=N1	1
Olopatadine Hydrochloride	CN(C)CC/C=C\1/C2=CC=CC=C2COC3=C1C=C(C=C3)CC(=O)O.Cl	1
Guanfacine	NC(=N)NC(=O)CC1=C(Cl)C=CC=C1Cl	1
Rosiglitazone Maleate	CN(CCOC1=CC=C(C=C1)CC2C(=O)NC(=O)S2)C3=CC=CC=N3	1
Bisoprolol Fumarate	CC(C)NCC(COC1=CC=C(C=C1)COCCOC(C)C)O	1
Donepezil Hydrochloride	COC1=C(C=C2C(=C1)CC(C2=O)CC3CCN(CC3)CC4=CC=CC=C4)OC.Cl	1
Ramipril	CCOC(=O)C(CCC1=CC=CC=C1)NC(C)C(=O)N2C3CCCC3CC2C(=O)O	1
Zolpidem Tartrate	CC1=CC=C(C=C1)C2=C(N3C=C(C=CC3=N2)C)CC(=O)N(C)C	1
Amlodipine	CCOC(=O)C1=C(NC(=C(C1C2=CC=CC=C2Cl)C(=O)OC)C)COCCN	1
Cetirizine	C1CN(CCN1CCOCC(=O)O)C(C2=CC=CC=C2)C3=CC=C(C=C3)Cl	1
Cyclobenzaprine	CN(C)CCC=C1C2=CC=CC=C2C=CC2=CC=CC=C12	1
Memantine	CC12CC3CC(C)(C1)CC(N)(C3)C2	1
Prednisolone	CC12CC(C3C(C1CCC2(C(=O)CO)O)CCC4=CC(=O)C=CC34C)O	1
Primaquine	CC(CCCN)NC1=C2C(=CC(=C1)OC)C=CC=N2	1
Diazepam	CN1C(=O)CN=C(C2=C1C=CC(=C2)Cl)C3=CC=CC=C3	1
Escitalopram Oxalate	CN(C)CCCC1(C2=C(CO1)C=C(C=C2)C#N)C3=CC=C(C=C3)F	1

Fluoxetine Hydrochloride	CNCCC(C1=CC=CC=C1)OC2=CC=C(C=C2)C(F)(F)F.Cl	1
Vitamin B6	CC1=NC=C(C(=C1O)CO)CO	1
Buspirone	O=C1CC2(CCCC2)CC(=O)N1CCCCN1CCN(CC1)C1=NC=CC=N1	1
Doxycycline Hyclate	CCO.CC1C2C(C3C(C(=O)C(=C(C3(C(=O)C2=C(C4=C1C=CC=C4O)O)O)O)C(=O)N)N(C)C)O.CC1C2C(C3C(C(=O)C(=C(C3(C(=O)C2=C(C4=C1C=CC=C4O)O)O)O)C(=O)N)N(C)C)O.O.Cl.Cl	1
Stavudine	CC1=CN(C(=O)NC1=O)[C@H]2C=C[C@H](O2)CO	1
Mirtazapine	CN1CCN2C(C1)C1=CC=CC=C1CC1=C2N=CC=C1	1
Cyclophosphamide	C1CNP(=O)(OC1)N(CCCl)CCCl	1
Prednisone	[H][C@@]12CC[C@](O)(C(=O)CO)[C@@]1(C)CC(=O)[C@@]1([H])[C@@]2([H])CCC2=CC(=O)C=C[C@]12C	1
Tramadol	COC1=CC=CC(=C1)C1(O)CCCCC1CN(C)C	1
Phenobarbital	CCC1(C(=O)NC(=O)NC1=O)C2=CC=CC=C2	1
Atomoxetine Hydrochloride	CC1=CC=CC=C1O[C@H](CCNC)C2=CC=CC=C2.Cl	1
Nortriptyline	CNCCC=C1C2=CC=CC=C2CCC2=CC=CC=C12	1
Diphenhydramine	CN(C)CCOC(C1=CC=CC=C1)C1=CC=CC=C1	1
Propranolol Hydrochloride	CC(C)NCC(COC1=CC=CC2=CC=CC=C21)O.Cl	1
Benznidazole	C1=CC=C(C=C1)CNC(=O)CN2C=CN=C2[N+](=O)[O-]	1
Diethylcarbamazine Citrate	CCN(CC)C(=O)N1CCN(CC1)C	1
Proguanil	CC(C)N=C(N)/N=C(\N)/NC1=CC=C(C=C1)Cl	1
Vitamin B2	CC1=CC2=C(C=C1C)N(C3=NC(=O)NC(=O)C3=N2)C[C@@H]([C@@H]([C@@H](CO)O)O)O	1
Meperidine	CCOC(=O)C1(CCN(C)CC1)C1=CC=CC=C1	1
Metoprolol Tartrate	CC(C)NCC(COC1=CC=C(C=C1)CCOC)O	1
Venlafaxine Hydrochloride	CN(C)CC(C1=CC=C(C=C1)OC)C2(CCCCC2)O.Cl	1
Bupropion	CC(NC(C)(C)C)C(=O)C1=CC(Cl)=CC=C1	1
Doxepin	[H]C(CCN(C)C)=C1C2=CC=CC=C2COC2=CC=CC=C12	1
Methionine	CSCCC(C(=O)O)N	1
Emtricitabine	C1C(OC(S1)CO)N2C=C(C(=NC2=O)N)F	1
Ethosuximide	CCC1(CC(=O)NC1=O)C	1
Mexiletine	CC(N)COC1=C(C)C=CC=C1C	1
Zidovudine	CC1=CN(C(=O)NC1=O)C2CC(C(O2)CO)N=[N+]=[N-]	1
Pregabalin	CC(C)CC(CC(=O)O)CN	1
Fluconazole	C1=CC(=C(C=C1F)F)C(CN2C=NC=N2)(CN3C=NC=N3)O	1
Chloroquine	CCN(CC)CCCC(C)NC1=C2C=CC(=CC2=NC=C1)Cl	1
Metronidazole	CC1=NC=C(N1CCO)[N+](=O)[O-]	1

Penicillin V	CC1([C@@H](N2[C@H](S1)[C@@H](C2=O)NC(=O)COC3=CC=CC=C3)C(=O)O)C	1
Flucytosine	NC1=C(F)C=NC(=O)N1	1
Theophylline Anhydrous	CN1C2=C(C(=O)N(C1=O)C)NC=N2	1
Niacinamide	C1=CC(=CN=C1)C(=O)N	1
Procainamide Hydrochloride	CCN(CC)CCNC(=O)C1=CC=C(C=C1)N.Cl	1
Potassium Chloride	[Cl-].[K+]	1
Lactulose	OC[C@H]1O[C@](O)(CO)[C@@H](O)[C@@H]1O[C@@H]1O[C@H](CO)[C@H](O)[C@H](O)[C@H]1O	2
Budesonide	[H][C@@]12C[C@H]3OC(CCC)O[C@@]3(C(=O)CO)[C@@]1(C)C[C@H](O)[C@@]1([H])[C@@]2([H])CCC2=CC(=O)C=C[C@]12C	2
Glimepiride	CCC1=C(CN(C1=O)C(=O)NCCC2=CC=C(C=C2)S(=O)(=O)NC(=O)NC3CCC(CC3)C)C	2
Risperidone	CC1=C(CCN2CCC(CC2)C2=NOC3=C2C=CC(F)=C3)C(=O)N2CCCCC2=N1	2
Triamcinolone	CC12CC(C3(C(C1CC(C2(C(=O)CO)O)O)CCC4=CC(=O)C=CC43C)F)O	2
Betamethasone	[H][C@@]12C[C@H](C)[C@](O)(C(=O)CO)[C@@]1(C)C[C@H](O)[C@@]1(F)[C@@]2([H])CCC2=CC(=O)C=C[C@]12C	2
Tacrolimus	CC1CC(C2C(CC(C(O2)(C(=O)C(=O)N3CCCCC3C(=O)OC(C(C(CC(=O)C(C=C(C1)C)CC=C)O)C)C(=CC4CCC(C(C4)OC)O)C)O)C)OC)OC	2
Ezetimibe	C1=CC(=CC=C1C2C(C(=O)N2C3=CC=C(C=C3)F)CCC(C4=CC=C(C=C4)F)O)O	2
Felodipine	CCOC(=O)C1=C(C)NC(C)=C(C1C1=C(Cl)C(Cl)=CC=C1)C(=O)OC	2
Loratadine	CCOC(=O)N1CCC(CC1)=C1C2=C(CCC3=C1N=CC=C3)C=C(Cl)C=C2	2
Nifedipine	CC1=C(C(C(=C(N1)C)C(=O)OC)C2=CC=CC=C2[N+](=O)[O-])C(=O)OC	2
Methylphenidate Hydrochloride	COC(=O)C(C1CCCCN1)C2=CC=CC=C2.Cl	2
Piroxicam	CN1C(C(=O)NC2=NC=CC=C2)=C(O)C2=C(C=CC=C2)S1(=O)=O	2
Indomethacin	COC1=CC2=C(C=C1)N(C(=O)C1=CC=C(Cl)C=C1)C(C)=C2CC(O)=O	2
Prochlorperazine	CN1CCN(CCCN2C3=CC=CC=C3SC3=C2C=C(Cl)C=C3)CC1	2
Aripiprazole	ClC1=CC=CC(N2CCN(CCCCOC3=CC4=C(CCC(=O)N4)C=C3)CC2)=C1Cl	2
Lansoprazole	CC1=C(OCC(F)(F)F)C=CN=C1CS(=O)C1=NC2=CC=CC=C2N1	2
Temazepam	CN1C2=C(C=C(Cl)C=C2)C(=NC(O)C1=O)C1=CC=CC=C1	2
Candesartan Cilexetil	CCOC1=NC2=C(N1CC1=CC=C(C=C1)C1=CC=CC=C1C1=NN=NN1)C(=CC=C2)C(=O)OC(C)OC(=O)OC1CCCCC1	2
Lovastatin	CCC(C)C(=O)OC1CC(C=C2C1C(C(C=C2)C)CCC3CC(CC(=O)O3)O)C	2
Olanzapine	CN1CCN(CC1)C1=NC2=CC=CC=C2NC2=C1C=C(C)S2	2
Omeprazole	COC1=CC2=C(C=C1)N=C(N2)S(=O)CC1=NC=C(C)C(OC)=C1C	2

Pioglitazone Hydrochloride	CCC1=CN=C(C=C1)CCOC2=CC=C(C=C2)CC3C(=O)NC(=O)S3.Cl	2
Amprenavir	CC(C)CN(CC(C(CC1=CC=CC=C1)NC(=O)OC2CCOC2)O)S(=O)(=O)C3=CC=C(C=C3)N	2
Clotrimazole	ClC1=CC=CC=C1C(N1C=CN=C1)(C1=CC=CC=C1)C1=CC=CC=C1	2
Diclofenac Sodium	C1=CC=C(C(=C1)CC(=O)[O-])NC2=C(C=CC=C2Cl)Cl.[Na+]	2
Meclizine	CC1=CC(CN2CCN(CC2)C(C2=CC=CC=C2)C2=CC=C(Cl)C=C2)=CC=C1	2
Simvastatin	CCC(C)(C)C(=O)OC1CC(C=C2C1C(C(C=C2)C)CCC3CC(CC(=O)O3)O)C	2
Telmisartan	CCCC1=NC2=C(C=C(C=C2C)C2=NC3=CC=CC=C3N2C)N1CC1=CC=C(C=C1)C1=CC=CC=C1C(O)=O	2
Ziprasidone Hydrochloride	C1CN(CCN1CCC2=C(C=C3C(=C2)CC(=O)N3)Cl)C4=NSC5=CC=CC=C54.Cl	2
Atorvastatin	CC(C)C1=C(C(=O)NC2=CC=CC=C2)C(=C(N1CC[C@@H](O)C[C@@H](O)CC(O)=O)C1=CC=C(F)C=C1)C1=CC=CC=C1	2
Nitrofurantoin	C1C(=O)NC(=O)N1/N=C/C2=CC=C(O2)[N+](=O)[O-]	2
Triamterene	NC1=NC(N)=C2N=C(C(N)=NC2=N1)C1=CC=CC=C1	2
Hydroxyzine	OCCOCCN1CCN(CC1)C(C1=CC=CC=C1)C1=CC=C(Cl)C=C1	2
Duloxetine	CNCC[C@H](OC1=CC=CC2=CC=CC=C12)C1=CC=CS1	2
Fenofibrate	CC(C)OC(=O)C(C)(C)OC1=CC=C(C=C1)C(=O)C1=CC=C(Cl)C=C1	2
Rifabutin	CC1C=CC=C(C(=O)N=C2C(=C3C(=C4C2=NC5(N4)CCN(CC5)CC(C)C)C6=C(C(=C3O)C)OC(C6=O)(OC=CC(C(C(C(C(C(C1O)C)O)C)OC(=O)C)C)OC)C)O)C	2
Nevirapine	CC1=C2C(=NC=C1)N(C3=C(C=CC=N3)C(=O)N2)C4CC4	2
Celecoxib	CC1=CC=C(C=C1)C1=CC(=NN1C1=CC=C(C=C1)S(N)(=O)=O)C(F)(F)F	2
Ketoconazole	CC(=O)N1CCN(CC1)C1=CC=C(OCC2COC(CN3C=CN=C3)(O2)C2=CC=C(Cl)C=C2Cl)C=C1	2
Lamotrigine	NC1=NC(N)=C(N=N1)C1=C(Cl)C(Cl)=CC=C1	2
Primidone	CCC1(C(=O)NCNC1=O)C1=CC=CC=C1	2
Phenytoin Sodium	C1=CC=C(C=C1)C2(C(=O)[N-]C(=O)N2)C3=CC=CC=C3.[Na+]	2
Clopidogrel Bisulfate	COC(=O)C(C1=CC=CC=C1Cl)N2CCC3=C(C2)C=CS3	2
Irbesartan	CCCCC1=NC2(CCCC2)C(=O)N1CC1=CC=C(C=C1)C1=CC=CC=C1C1=NNN=N1	2
Quetiapine Fumarate	C1CN(CCN1CCOCCO)C2=NC3=CC=CC=C3SC4=CC=CC=C42	2
Carbamazepine	C1=CC=C2C(=C1)C=CC3=CC=CC=C3N2C(=O)N	2
Loracarbef	C1CC(=C(N2C1C(C2=O)NC(=O)C(C3=CC=CC=C3)N)C(=O)O)Cl	2
Iopanoic Acid	CCC(CC1=C(C(=C(C=C1I)I)N)I)C(=O)O	2
Mycophenolate Mofetil	COC1=C(C\C=C(/C)CCC(=O)OCCN2CCOCC2)C(O)=C2C(=O)OCC2=C1C	2
Tipranavir	CCCC1(CC(=C(C(=O)O1)C(CC)C2=CC(=CC=C2)NS(=O)(=O)C3=NC=C(C=C3)C(F)(F)F)O)CCC4=CC=CC=C4	2

Praziquantel	C1CCC(CC1)C(=O)N2CC3C4=CC=CC=C4CCN3C(=O)C2	2
Rifampin	C[C@H]1/C=C/C=C(\C(=O)NC2=C(C(=C3C(=C2O)C(=C(C4=C3C(=O)[C@](O4)(O/C=C/[C@@H]([C@H]([C@H]([C@@H]([C@@H]([C@@H]([C@H]1O)C)O)C)OC(=O)C)C)OC)C)C)O)O)/C=N/N5CCN(CC5)C)/C	2
Etodolac	CCC1=C2NC3=C(CCOC3(CC)CC(O)=O)C2=CC=C1	2
Felbamate	NC(=O)OCC(COC(N)=O)C1=CC=CC=C1	2
Gemfibrozil	CC1=CC(OCCCC(C)(C)C(O)=O)=C(C)C=C1	2
Oxaprozin	OC(=O)CCC1=NC(=C(O1)C1=CC=CC=C1)C1=CC=CC=C1	2
Hydroxychloroquine	CCN(CCO)CCCC(C)NC1=C2C=CC(Cl)=CC2=NC=C1	2
Mycophenolic Acid	COC1=C(C\C=C(/C)CCC(O)=O)C(O)=C2C(=O)OCC2=C1C	2
Atovaquone	OC1=C([C@H]2CC[C@@H](CC2)C2=CC=C(Cl)C=C2)C(=O)C2=CC=CC=C2C1=O	2
Naproxen	COC1=CC2=C(C=C1)C=C(C=C2)[C@H](C)C(O)=O	2
Ibuprofen	CC(C)CC1=CC=C(C=C1)C(C)C(=O)O	2
Sulfamethoxazole	CC1=CC(=NO1)NS(=O)(=O)C2=CC=C(C=C2)N	2
Metaxalone	CC1=CC(OCC2CNC(=O)O2)=CC(C)=C1	2
Nalidixic Acid	CCN1C=C(C(=O)C2=C1N=C(C=C2)C)C(=O)O	2
Fosamprenavir	CC(C)CN(CC(C(CC1=CC=CC=C1)NC(=O)OC2CCOC2)OP(=O)(O)O)S(=O)(=O)C3=CC=C(C=C3)N	2
Ergonovine	C[C@@H](CO)NC(=O)[C@H]1CN([C@@H]2CC3=CNC4=CC=CC(=C34)C2=C1)C	3
Reserpine	CO[C@H]1[C@@H](C[C@@H]2CN3CCC4=C([C@H]3C[C@@H]2[C@@H]1C(=O)OC)NC5=C4C=CC(=C5)OC)OC(=O)C6=CC(=C(C(=C6)OC)OC)OC	3
Colchicine	CC(=O)N[C@H]1CCC2=CC(=C(C(=C2C3=CC=C(C(=O)C=C13)OC)OC)OC)OC	3
Vitamin D2	C[C@H](/C=C/[C@H](C)C(C)C)[C@H]1CC[C@@H]\2[C@@]1(CCC/C2=C\C=C/3\C[C@H](CCC3=C)O)C	3
Biperiden	C1CCN(CC1)CCC(C2CC3CC2C=C3)(C4=CC=CC=C4)O	3
Ergotamine Tartrate	CC1(C(=O)N2C(C(=O)N3CCCC3C2(O1)O)CC4=CC=CC=C4)NC(=O)C5CN(C6CC7=CNC8=CC=CC(=C78)C6=C5)C	3
Naloxone	OC1=CC=C2C[C@H]3N(CC=C)CC[C@@]45[C@@H](OC1=C24)C(=O)CC[C@@]35O	3
Levocetirizine	OC(=O)COCCN1CCN(CC1)[C@H](C1=CC=CC=C1)C1=CC=C(Cl)C=C1	3
Thyroxine	C1=C(C=C(C(=C1I)OC2=CC(=C(C(=C2)I)O)I)I)C[C@@H](C(=O)O)N	3
Ondansetron Hydrochloride	CC1=NC=CN1CC2CCC3=C(C2=O)C4=CC=CC=C4N3C.Cl	3
Amlodipine Besylate	CCOC(=O)C1=C(NC(=C(C1C2=CC=CC=C2Cl)C(=O)OC)C)COCCN.C1=CC=C(C=C1)S(=O)(=O)O	3
Minoxidil	NC1=CC(=NC(N)=[N+]1[O-])N1CCCCC1	3

Neostigmine Methylsulfate	CN(C)C(=O)OC1=CC=CC(=C1)[N+](C)(C)C	3
Methotrexate	CN(CC1=CN=C2C(=N1)C(=NC(=N2)N)N)C3=CC=C(C=C3)C(=O)N[C@@H](CCC(=O)O)C(=O)O	3
Pyridostigmine Bromide	C[N+]1=CC=CC(=C1)OC(=O)N(C)C	3
Famotidine	NC(N)=NC1=NC(CSCCC(N)=NS(N)(=O)=O)=CS1	3
Lisinopril	NCCCC[C@H](N[C@@H](CCC1=CC=CC=C1)C(O)=O)C(=O)N1CCC[C@H]1C(O)=O	3
Hydrochlorothiazide	C1NC2=CC(=C(C=C2S(=O)(=O)N1)S(=O)(=O)N)Cl	3
Propylthiouracil	CCCC1=CC(=O)NC(=S)N1	3
Alendronate Sodium	C(CC(O)(P(=O)(O)O)P(=O)(O)[O-])CN.[Na+]	3
Oseltamivir Phosphate	CCC(CC)O[C@@H]1C=C(C[C@@H]([C@H]1NC(=O)C)N)C(=O)OCC.OP(=O)(O)O	3
Nadolol	CC(C)(C)NCC(O)COC1=CC=CC2=C1C[C@H](O)[C@H](O)C2	3
Pravastatin Sodium	CC[C@H](C)C(=O)O[C@H]1C[C@@H](C=C2[C@H]1[C@H]([C@H](C=C2)C)CC[C@H](C[C@H](CC(=O)[O-])O)O)O.[Na+]	3
Atenolol	CC(C)NCC(COC1=CC=C(C=C1)CC(=O)N)O	3
Hydralazine Hydrochloride	C1=CC=C2C(=C1)C=NN=C2NN.Cl	3
Sumatriptan Succinate	CNS(=O)(=O)CC1=CC2=C(C=C1)NC=C2CCN(C)C.C(CC(=O)O)C(=O)O	3
Hydralazine	NNC1=NN=CC2=CC=CC=C12	3
Pseudoephedrine	CN[C@@H](C)[C@@H](O)C1=CC=CC=C1	3
Risedronate Sodium	C1=CC(=CN=C1)CC(O)(P(=O)(O)O)P(=O)(O)[O-].[Na+]	3
Benzonatate	CCCCNC1=CC=C(C=C1)C(=O)OCCOCCOCCOCCOCCOCCOCCOCCOC	3
Ribavirin	NC(=O)C1=NN(C=N1)[C@@H]1O[C@H](CO)[C@@H](O)[C@H]1O	3
Topiramate	[H][C@@]12CO[C@@]3(COS(N)(=O)=O)OC(C)(C)O[C@@]3([H])[C@]1([H])OC(C)(C)O2	3
Chloramphenicol	C1=CC(=CC=C1[C@H]([C@@H](CO)NC(=O)C(Cl)Cl)O)[N+](=O)[O-]	3
Didanosine	C1C[C@@H](O[C@@H]1CO)N2C=NC3=C2N=CNC3=O	3
Penicillamine	CC(C)([C@H](C(=O)O)N)S	3
Abacavir Sulfate	C1CC1NC2=C3C(=NC(=N2)N)N(C=N3)[C@@H]4C[C@@H](C=C4)CO.C1CC1NC2=C3C(=NC(=N2)N)N(C=N3)[C@@H]4C[C@@H](C=C4)CO.OS(=O)(=O)O	3
Allopurinol	C1=NNC2=C1C(=O)NC=N2	3
Ranitidine Hydrochloride	CN/C(=C\[N+](=O)[O-])/NCCSCC1=CC=C(O1)CN(C)C.Cl	3

Ethambutol Hydrochloride	CC[C@@H](CO)NCCN[C@@H](CC)CO.Cl.Cl	3
Valganciclovir	CC(C)[C@H](N)C(=O)OCC(CO)OCN1C=NC2=C1NC(N)=NC2=O	3
Erythromycin	CC[C@@H]1[C@@]([C@@H]([C@H](C(=O)[C@@H](C[C@@]([C@@H]([C@H]([C@@H]([C@H](C(=O)O1)C)O[C@H]2C[C@@]([C@H]([C@@H](O2)C)O)(C)OC)C)O[C@H]3[C@@H]([C@H](C[C@H](O3)C)N(C)C)O)(C)O)C)C)O)(C)O	3
Amoxicillin Trihydrate	CC1([C@@H](N2[C@H](S1)[C@@H](C2=O)NC(=O)[C@@H](C3=CC=C(C=C3)O)N)C(=O)O)C.O.O.O	3
Methyldopa	C[C@](CC1=CC(=C(C=C1)O)O)(C(=O)O)N	3
Vitamin B1	CC1=C(SC=[N+]1CC2=CN=C(N=C2N)C)CCO.[Cl-]	3
Cefaclor	[H][C@]12SCC(Cl)=C(N1C(=O)[C@H]2NC(=O)[C@H](N)C1=CC=CC=C1)C(O)=O	3
Cefprozil	[H][C@]12SCC(C=CC)=C(N1C(=O)[C@@]2([H])NC(=O)[C@H](N)C1=CC=C(O)C=C1)C(O)=O	3
Acyclovir Sodium	C1=NC2=C(N1COCCO)N=C(N=C2[O-])N.[Na+]	3
Cimetidine	CC1=C(N=CN1)CSCCNC(=NC)NC#N	3
Gabapentin	NCC1(CC(O)=O)CCCCC1	3
Metformin	CN(C)C(=N)NC(N)=N	3
Acetaminophen	CC(=O)NC1=CC=C(C=C1)O	3
Cloxacillin	CC1=C(C(=NO1)C2=CC=CC=C2Cl)C(=O)N[C@H]3[C@@H]4N(C3=O)[C@H](C(S4)(C)C)C(=O)O	3
Erythromycin Lactobionate	CC[C@@H]1[C@@]([C@@H]([C@H](C(=O)[C@@H](C[C@@]([C@@H]([C@H]([C@@H]([C@H](C(=O)O1)C)O[C@H]2C[C@@]([C@H]([C@@H](O2)C)O)(C)OC)C)O[C@H]3[C@@H]([C@H](C[C@H](O3)C)N(C)C)O)(C)O)C)C)O)(C)O.C([C@@H]1[C@@H]([C@@H]([C@H]([C@@H](O1)O[C@H]([C@@H](CO)O)[C@@H]([C@H](C(=O)O)O)O)O)O)O)O	3
Metformin Hydrochloride	CN(C)C(=N)N=C(N)N.Cl	3
Vitamin C	C([C@@H]([C@@H]1C(=C(C(=O)O1)O)O)O)O	3
Cefadroxil	[H][C@]12SCC(C)=C(N1C(=O)[C@H]2NC(=O)[C@H](N)C1=CC=C(O)C=C1)C(O)=O	3
Loperamide	CN(C)C(=O)C(CCN1CCC(O)(CC1)C1=CC=C(Cl)C=C1)(C1=CC=CC=C1)C1=CC=CC=C1	4
Glipizide	CC1=NC=C(N=C1)C(=O)NCCC1=CC=C(C=C1)S(=O)(=O)NC(=O)NC1CCCCC1	4
Triamcinolone Acetonide	CC1(OC2CC3C4CCC5=CC(=O)C=CC5(C4(C(CC3(C2(O1)C(=O)CO)C)O)F)C)C	4
Chlorthalidone	NS(=O)(=O)C1=C(Cl)C=CC(=C1)C1(O)NC(=O)C2=CC=CC=C12	4
Etoposide	[H][C@]12COC(=O)[C@]1([H])[C@H](C1=CC(OC)=C(O)C(OC)=C1)C1=CC3=C(OCO3)C=C1[C@H]2O[C@@H]1O[C@]2([H])CO[C@@H](C)O[C@@]2([H])[C@H](O)[C@H]1O	4

Mercaptopurine	S=C1N=CNC2=C1NC=N2	4
Azathioprine	CN1C=NC(=C1SC2=NC=NC3=C2NC=N3)[N+](=O)[O-]	4
Furosemide	C1=COC(=C1)CNC2=CC(=C(C=C2C(=O)O)S(=O)(=O)N)Cl	4
Cefdinir	[H][C@]12SCC(C=C)=C(N1C(=O)[C@H]2NC(=O)C(=N/O)\C1=CSC(N)=N1)C(O)=O	4
Acetazolamide	CC(=O)NC1=NN=C(S1)S(=O)(=O)N	4
Saquinavir Methanesulfonate	CC(C)(C)NC(=O)[C@@H]1C[C@@H]2CCCC[C@@H]2CN1C[C@H]([C@H](CC3=CC=CC=C3)NC(=O)[C@H](CC(=O)N)NC(=O)C4=NC5=CC=CC=C5C=C4)O.CS(=O)(=O)[O-]	4
Cefixime	[H][C@]12SCC(C=C)=C(N1C(=O)[C@H]2NC(=O)C(=N/OCC(O)=O)\C1=CSC(N)=N1)C(O)=O	4
Clarithromycin	[H][C@@]1(C[C@@](C)(OC)[C@@H](O)[C@H](C)O1)O[C@H]1[C@H](C)[C@@H](O[C@]2([H])O[C@H](C)C[C@@H]([C@H]2O)N(C)C)[C@@](C)(C[C@@H](C)C(=O)[C@H](C)[C@@H](O)[C@](C)(O)[C@@H](CC)OC(=O)[C@@H]1C)OC	4
Linezolid	CC(=O)NC[C@H]1CN(C(=O)O1)C1=CC(F)=C(C=C1)N1CCOCC1	4
Nelfinavir	CC1=C(C=CC=C1O)C(=O)N[C@@H](CSC2=CC=CC=C2)[C@@H](CN3C[C@H]4CCCC[C@H]4C[C@H]3C(=O)NC(C)(C)C)O	4
Cephalexin	[H][C@]12SCC(C)=C(N1C(=O)[C@H]2NC(=O)[C@H](N)C1=CC=CC=C1)C(O)=O	4
Mesalamine	C1=CC(=C(C=C1N)C(=O)O)O	4
Chlorothiazide	NS(=O)(=O)C1=C(Cl)C=C2NC=NS(=O)(=O)C2=C1	4
Digoxin	C[C@@H]1[C@H]([C@H](C[C@@H](O1)O[C@@H]2[C@H](O[C@H](C[C@@H]2O)O[C@@H]3[C@H](O[C@H](C[C@@H]3O)O[C@H]4CC[C@]5([C@@H](C4)CC[C@@H]6[C@@H]5C[C@H]([C@]7([C@@]6(CC[C@@H]7C8=CC(=O)OC8)O)C)O)C)C)C)O)O	1,2
Flecainide	FC(F)(F)COC1=CC(C(=O)NCC2CCCCN2)=C(OCC(F)(F)F)C=C1	1,2
Warfarin	CC(=O)CC(C1=CC=CC=C1)C2=C(C3=CC=CC=C3OC2=O)O	1,2
Amitriptyline Hydrochloride	CN(C)CCC=C1C2=CC=CC=C2CCC3=CC=CC=C31.Cl	1,2
Imipramine	CN(C)CCCN1C2=CC=CC=C2CCC2=CC=CC=C12	1,2
Verapamil Hydrochloride	CC(C)C(CCCN(C)CCC1=CC(=C(C=C1)OC)OC)(C#N)C2=CC(=C(C=C2)OC)OC.Cl	1,2
Sertraline Hydrochloride	CN[C@H]1CC[C@H](C2=CC=CC=C12)C3=CC(=C(C=C3)Cl)Cl.Cl	1,2
Finasteride	[H][C@@]12CC[C@H](C(=O)NC(C)(C)C)[C@@]1(C)CC[C@@]1([H])[C@@]2([H])CC[C@@]2([H])NC(=O)C=C[C@]12C	1,2
Montelukast Sodium	CC(C)(C1=CC=CC=C1CC[C@H](C2=CC=CC(=C2)/C=C/C3=NC4=C(C=CC(=C4)Cl)C=C3)SCC5(CC5)CC(=O)[O-])O.[Na+]	1,2
Doxazosin Mesylate	COC1=C(C=C2C(=C1)C(=NC(=N2)N3CCN(CC3)C(=O)C4COC5=CC=CC=C5O4)N)OC.CS(=O)(=O)O	1,2

Terbinafine Hydrochloride	CC(C)(C)C#C/C=C/CN(C)CC1=CC=CC2=CC=CC=C21.Cl	1,2
Valproic Acid	CCCC(CCC)C(=O)O	1,2
Fosinopril	CCC(=O)O[C@@H](OP(=O)(CCCCC1=CC=CC=C1)CC(=O)N1C[C@@H](C[C@H]1C(O)=O)C1CCCCC1)C(C)C	1,3
Promethazine Hydrochloride	CC(CN1C2=CC=CC=C2SC3=CC=CC=C31)N(C)C.Cl	1,3
Carbidopa	C[C@@](CC1=CC(O)=C(O)C=C1)(NN)C(O)=O	1,3
Levodopa	C1=CC(=C(C=C1C[C@@H](C(=O)O)N)O)O	1,3
Isoniazid	C1=CN=CC=C1C(=O)NN	1,3
Albuterol Sulfate	CC(C)(C)NCC(C1=CC(=C(C=C1)O)CO)O.CC(C)(C)NCC(C1=CC(=C(C=C1)O)CO)O.OS(=O)(=O)O	1,3
Pyrazinamide	C1=CN=C(C=N1)C(=O)N	1,3
Atropine Sulfate	CN1[C@@H]2CC[C@H]1CC(C2)OC(=O)C(CO)C3=CC=CC=C3.CN1[C@@H]2CC[C@H]1CC(C2)OC(=O)C(CO)C3=CC=CC=C3.OS(=O)(=O)O	1,3
Nitroglycerin	C(C(CO[N+](=O)[O-])O[N+](=O)[O-])O[N+](=O)[O-]	1,3
Ethinylestradiol	C[C@]12CC[C@H]3[C@H]([C@@H]1CC[C@]2(C#C)O)CCC4=C3C=CC(=C4)O	1,3
Codeine Monohydrate	CN1CC[C@]23[C@@H]4[C@H]1CC5=C2C(=C(C=C5)OC)O[C@H]3[C@H](C=C4)O.O	1,3
Codeine	[H][C@]12C=C[C@H](O)[C@@H]3OC4=C5C(C[C@H]1N(C)CC[C@@]235)=CC=C4OC	1,3
Caffeine	CN1C=NC2=C1C(=O)N(C)C(=O)N2C	1,3
Captopril	C[C@H](CS)C(=O)N1CCC[C@H]1C(=O)O	1,3
Levetiracetam	CC[C@H](N1CCCC1=O)C(N)=O	1,3
Niacin	OC(=O)C1=CN=CC=C1	1,3
Chlorpheniramine Maleate	CN(C)CCC(C1=CC=C(C=C1)Cl)C2=CC=CC=N2.C(=C\C(=O)O)\C(=O)O	1,3
Dexamethasone	C[C@@H]1C[C@H]2[C@@H]3CCC4=CC(=O)C=C[C@@]4([C@]3([C@H](C[C@@]2([C@]1(C(=O)CO)O)C)O)F)C	1,3
Metoclopramide Hydrochloride	CCN(CC)CCNC(=O)C1=CC(=C(C=C1OC)N)Cl.Cl	1,3
Amoxicillin	CC1(C(N2C(S1)C(C2=O)NC(=O)C(C3=CC=C(C=C3)O)N)C(=O)O)C	1,3
Morphine Hydrochloride	CN1CC[C@]23[C@@H]4[C@H]1CC5=C2C(=C(C=C5)O)O[C@H]3[C@H](C=C4)O.Cl	1,3
Cetirizine Hydrochloride	C1CN(CCN1CCOCC(=O)O)C(C2=CC=CC=C2)C3=CC=C(C=C3)Cl.Cl.Cl	1,3
Morphine	[H][C@@]12OC3=C(O)C=CC4=C3[C@@]11CCN(C)[C@]([H])(C4)[C@]1([H])C=C[C@@H]2O	1,3
Fexofenadine	CC(C)(C(O)=O)C1=CC=C(C=C1)C(O)CCCN1CCC(CC1)C(O)(C1=CC=CC=C1)C1=CC=CC=C1	1,3

Losartan Potassium	CCCCC1=NC(=C(N1CC2=CC=C(C=C2)C3=CC=CC=C3C4=NN=N[N-]4)CO)Cl.[K+]	1,3
Clindamycin	CCCC1CC(N(C1)C)C(=O)NC(C2C(C(C(C(O2)SC)O)O)O)C(C)Cl	1,3
Isosorbide Dinitrate	C1[C@H]([C@@H]2[C@H](O1)[C@H](CO2)O[N+](=O)[O-])O[N+](=O)[O-]	1,3
Clomiphene Citrate	CCN(CC)CCOC1=CC=C(C=C1)/C(=C(/C2=CC=CC=C2)\Cl)/C3=CC=CC=C3.C(C(=O)O)C(CC(=O)O)(C(=O)O)O	1,3
Levamisole	C1CSC2=N[C@H](CN21)C3=CC=CC=C3	1,3
Acetylsalicylic Acid	CC(=O)OC1=CC=CC=C1C(=O)O	1,3
Doxycycline	[H][C@@]12[C@@H](C)C3=CC=CC(O)=C3C(=O)C1=C(O)[C@]1(O)C(=O)C(C(N)=O)=C(O)[C@@H](N(C)C)[C@]1([H])[C@H]2O	1,3
Levofloxacin	C[C@H]1COC2=C3N1C=C(C(O)=O)C(=O)C3=CC(F)=C2N1CCN(C)CC1	1,3
Clomipramine	CN(C)CCCN1C2=CC=CC=C2CCC3=C1C=C(C=C3)Cl	1,3
Quinine Bisulfate Heptahydrate	COC1=CC2=C(C=CN=C2C=C1)[C@H]([C@@H]3C[C@@H]4CCN3C[C@@H]4C=C)O.O.O.O.O.O.O.O.OS(=O)(=O)O	1,3
Lamivudine	C1[C@H](O[C@H](S1)CO)N2C=CC(=NC2=O)N	1,3
Clavulanic Acid	[H][C@@]12CC(=O)N1[C@@H](C(O)=O)\C(O2)=C\CO	1,3
Methylprednisolone	[H][C@@]12CC[C@](O)(C(=O)CO)[C@@]1(C)C[C@H](O)[C@@]1([H])[C@@]2([H])C[C@H](C)C2=CC(=O)C=C[C@]12C	2,4
Dapsone	C1=CC(=CC=C1N)S(=O)(=O)C2=CC=C(C=C2)N	2,4
Indinavir Sulfate	CC(C)(C)NC(=O)[C@@H]1CN(CCN1C[C@H](C[C@@H](CC2=CC=CC=C2)C(=O)N[C@@H]3[C@@H](CC4=CC=CC=C34)O)O)CC5=CN=CC=C5.OS(=O)(=O)O	2,4
Trimethoprim	COC1=CC(=CC(=C1OC)OC)CC2=CN=C(N=C2N)N	2,4
Ritonavir	CC(C)C1=NC(=CS1)CN(C)C(=O)N[C@@H](C(C)C)C(=O)N[C@@H](CC2=CC=CC=C2)C[C@@H]([C@H](CC3=CC=CC=C3)NC(=O)OCC4=CN=CS4)O	2,4
Griseofulvin	C[C@@H]1CC(=O)C=C([C@]12C(=O)C3=C(O2)C(=C(C=C3OC)OC)Cl)OC	2,4
Azithromycin	CC[C@H]1OC(=O)[C@H](C)[C@@H](O[C@H]2C[C@@](C)(OC)[C@@H](O)[C@H](C)O2)[C@H](C)[C@@H](O[C@@H]2O[C@H](C)C[C@@H]([C@H]2O)N(C)C)[C@](C)(O)C[C@@H](C)CN(C)[C@H](C)[C@@H](O)[C@]1(C)O	2,4
Dutasteride	[H][C@@]1(CC[C@@]2([H])[C@]3([H])CC[C@@]4([H])NC(=O)C=C[C@]4(C)[C@@]3([H])CC[C@]12C)C(=O)NC1=CC(=CC=C1C(F)(F)F)C(F)(F)F	2,4
Isradipine	COC(=O)C1=C(C)NC(C)=C(C1C1=CC=CC2=NON=C12)C(=O)OC(C)C	2,4
Dronabinol	[H][C@@]12C=C(C)CC[C@@]1([H])C(C)(C)OC1=C2C(O)=CC(CCCCC)=C1	2,4
Dicyclomine Hydrochloride	CCN(CC)CCOC(=O)C1(CCCCC1)C2CCCCC2.Cl	2,4

Carisoprodol	CCCC(C)(COC(N)=O)COC(=O)NC(C)C	2,4
Nabumetone	COC1=CC2=C(C=C1)C=C(CCC(C)=O)C=C2	2,4
Ivermectin	CC[C@H](C)[C@@H]1[C@H](CC[C@@]2(O1)C[C@@H]3C[C@H](O2)C/C=C(/[C@H]([C@H](/C=C/C=C/4\CO[C@H]5[C@@]4([C@@H](C=C([C@H]5O)C)C(=O)O3)O)C)O[C@H]6C[C@@H]([C@H]([C@@H](O6)C)O[C@H]7C[C@@H]([C@H]([C@@H](O7)C)O)OC)OC)\C)C	2,4
Glyburide	COC1=C(C=C(C=C1)Cl)C(=O)NCCC2=CC=C(C=C2)S(=O)(=O)NC(=O)NC3CCCCC3	2,4
Haloperidol	C1CN(CCC1(C2=CC=C(C=C2)Cl)O)CCCC(=O)C3=CC=C(C=C3)F	2,4
Clofazimine	CC(C)N=C1C=C2C(=NC3=CC=CC=C3N2C4=CC=C(C=C4)Cl)C=C1NC5=CC=C(C=C5)Cl	2,4
Mebendazole	COC(=O)NC1=NC2=C(N1)C=C(C=C2)C(=O)C3=CC=CC=C3	2,4
Vitamin A	CC1=C(C(CCC1)(C)C)/C=C/C(=C/C=C/C(=C/CO)/C)/C	2,4
Pyrantel Pamoate	CN1CCCN=C1/C=C/C2=CC=CS2.C1=CC=C2C(=C1)C=C(C(=C2CC3=C(C(=CC4=CC=CC=C43)C(=O)O)O)O)C(=O)O	2,4
Albendazole	CCCSC1=CC2=C(C=C1)N=C(N2)NC(=O)OC	2,4
Chlorpromazine Hydrochloride	CN(C)CCCN1C2=CC=CC=C2SC3=C1C=C(C=C3)Cl.Cl	2,4
Lopinavir	CC1=C(C(=CC=C1)C)OCC(=O)N[C@@H](CC2=CC=CC=C2)[C@H](C[C@H](CC3=CC=CC=C3)NC(=O)[C@H](C(C)C)N4CCCNC4=O)O	2,4
Mefloquine	C1CCNC(C1)C(C2=CC(=NC3=C2C=CC=C3C(F)(F)F)C(F)(F)F)O	2,4
Triclabendazole	CSC1=NC2=CC(=C(C=C2N1)Cl)OC3=C(C(=CC=C3)Cl)Cl	2,4
Diloxanide Furoate	CN(C1=CC=C(C=C1)OC(=O)C2=CC=CO2)C(=O)C(Cl)Cl	2,4
Niclosamide	C1=CC(=C(C=C1[N+](=O)[O-])Cl)NC(=O)C2=C(C=CC(=C2)Cl)O	2,4
Sulfadiazine	C1=CN=C(N=C1)NS(=O)(=O)C2=CC=C(C=C2)N	2,4
Calcitriol	C[C@H](CCCC(C)(C)O)[C@@]1([H])CC[C@@]2([H])\C(CCC[C@]12C)=C\C=C1\C[C@@H](O)C[C@H](O)C1=C	2,4
Modafinil	NC(=O)CS(=O)C(C1=CC=CC=C1)C1=CC=CC=C1	2,4
Progesterone	[H][C@@]12CC[C@H](C(C)=O)[C@@]1(C)CC[C@@]1([H])[C@@]2([H])CCC2=CC(=O)CC[C@]12C	2,4
Oxcarbazepine	NC(=O)N1C2=CC=CC=C2CC(=O)C2=C1C=CC=C2	2,4
Sulfasalazine	C1=CC=NC(=C1)NS(=O)(=O)C2=CC=C(C=C2)N=NC3=CC(=C(C=C3)O)C(=O)O	2,4
Meloxicam	CN1C(C(=O)NC2=NC=C(C)S2)=C(O)C2=C(C=CC=C2)S1(=O)=O	2,4
Tadalafil	[H][C@]12CC3=C(NC4=CC=CC=C34)[C@H](N1C(=O)CN(C)C2=O)C1=CC2=C(OCO2)C=C1	2,4
Efavirenz	C1CC1C#C[C@]2(C3=C(C=CC(=C3)Cl)NC(=O)O2)C(F)(F)F	2,4
Erythromycin Stearate	CCCCCCCCCCCCCCCCCC(=O)O.CC[C@@H]1[C@@]([C@@H]([C@H](C(=O)[C@@H](C[C@@]([C@@H]([C@H]([C@@H]([C@H](C(=O)O1)C)O[C@H]2C[C@@]([C@H]([C@@H](O2)C)O)(C)OC)C)O[C@H]3[C@@H]([C@H](C[C@H](O3)C)N(C)C)O)(C)O)C)C)O)(C)O	3,4
Ciprofloxacin	OC(=O)C1=CN(C2CC2)C2=CC(N3CCNCC3)=C(F)C=C2C1=O	3,4

Nystatin	C[C@H]1/C=C/C=C/CC/C=C/C=C/C=C/C=C/C(CC2C(C(C[C@](O2)(CC(C(CCC(CC(CC(CC(=O)O[C@H]([C@@H]([C@@H]1O)C)C)O)O)O)O)O)O)O)C(=O)O)O[C@@H]3[C@H]([C@H]([C@@H]([C@H](O3)C)O)N)O	3,4
Acyclovir	NC1=NC(=O)C2=C(N1)N(COCCO)C=N2	3,4
